# Harnessing the diversity of a lettuce wild relative to identify anthocyanin-related genes transcriptionally responsive to drought stress

**DOI:** 10.3389/fpls.2024.1494339

**Published:** 2025-01-15

**Authors:** Inés Medina-Lozano, Jérôme Grimplet, Aurora Díaz

**Affiliations:** ^1^ Department of Plant Sciences, Agrifood Research and Technology Centre of Aragon (CITA), Zaragoza, Spain; ^2^ AgriFood Institute of Aragon – IA2 (CITA-University of Zaragoza), Zaragoza, Spain

**Keywords:** abiotic stress, antioxidants, crop wild relatives, differentially expressed genes, *Lactuca sativa* L., real-time qPCR, RNA-seq, resilience

## Abstract

Lettuce is a crop particularly vulnerable to drought. A transcriptomic study in the variety ‘Romired’ and the wild relative *Lactuca homblei* was conducted to understand the increase in anthocyanins (only significant in *L. homblei*) in response to drought previously observed. RNA-seq revealed more differentially expressed genes (DEGs), especially upregulated, in the wild species, in which the most abundant and significant GO terms were involved in regulatory processes (including response to water). Anthocyanin synthesis was triggered in *L. homblei* in response to drought, with 17 genes activated out of the 36 mapped in the phenylpropanoid-flavonoid pathway compared to 7 in ‘Romired’. Nineteen candidate DEGs with the strongest change in expression and correlation with both anthocyanin content and drought were selected and validated by qPCR, all being differentially expressed only in the wild species with the two techniques. Their functions were related to anthocyanins and/or stress response and they harboured 404 and 11 polymorphisms in the wild and cultivated species, respectively. Some wild variants had high or moderate predicted impacts on the respective protein function: a transcription factor that responds to abiotic stresses, a heat shock protein involved in stomatal closure, and a phospholipase participating in anthocyanin accumulation under abiotic stress. These genetic variants could explain the differences in the gene expression patterns between the wild (significantly up/downregulated) and the cultivated (no significant changes) species. The diversity of this crop wild relative for anthocyanin-related genes involved in the response to drought could be exploited to improve lettuce resilience against some adverse climate effects.

## 1 Introduction

Abiotic stress is a major challenge for agriculture, especially in the present scenario of climate change (IPCC, 2021), in which adverse environmental conditions are more and more frequent (FAO, 2021). In particular, drought is one of the most concerning abiotic stresses, affecting both crop yield and quality. Drought stress has important effects on plant growth by affecting diverse physiological and biochemical processes, like cell expansion and photosynthesis due to stomatal closure (Farooq et al., 2009). Apart from biomass production, it also affects nutrient composition and concentration as well as secondary metabolism, depending generally on the stress severity and duration, as well as on plant tolerance (Reddy et al., 2004; Medina-Lozano et al., 2024).

Lettuce (*Lactuca sativa* L.) is one of the most important leafy vegetables worldwide (FAOSTAT, 2021). It provides different health benefits attributed to phenolic compound, vitamin, and fibre contents (Llorach et al., 2008), among others, what contributes to increase its popularity especially with the growing awareness of the impact of diet on health among consumers. Lettuce is mostly composed by water (up to 97%) (Mou, 2005), what makes it highly susceptible to drought (Eriksen et al., 2016). However, controlled deficit irrigation can cause an improvement of its health-promoting properties by increasing the content of some antioxidants (Paim et al., 2020; Medina-Lozano et al., 2024). Among the phenolic compounds present in lettuce, anthocyanins are responsible for red pigmentation of the leaves in semi-red and red varieties. They are known to play crucial roles in human health due to their antioxidant properties (Garcia and Blesso, 2021). It has been described that water stress causes an accumulation of anthocyanins in some fruits, vegetables and oil crops, such as grapes (Ju et al., 2019), strawberries (Rugienius et al., 2021), and purple-stem canola (Chen et al., 2022b). In lettuce, different studies had reported increased levels of either total phenolic compounds under water stress (Zeljković et al., 2023), or anthocyanins in response to other environmental stresses, like UV irradiance (Tsormpatsidis et al., 2008) and low temperatures (Becker et al., 2014). However, anthocyanin response to drought conditions had barely been studied in this crop until recently, when a drought-induced anthocyanin accumulation not only in cultivated lettuce varieties but also in wild relative species, has been discovered (Medina-Lozano et al., 2024).

Lettuce anthocyanin content is very dependent on the genotype. In absence of stress, commercial varieties are the richest, followed by traditional ones and finally by lettuce wild relatives (Medina-Lozano et al., 2021). Interestingly, in all the lettuce-related germplasm studied, the water stress always resulted in an increase of the total anthocyanin content, with the highest accumulation detected in a wild relative species (Medina-Lozano et al., 2024). Crop wild relatives (CWR) are known to be a source of favourable alleles for interesting traits for breeding, like resistance to diseases or tolerance to abiotic stresses (Quezada-Martinez et al., 2021).

Unveiling the molecular mechanisms governing the changes of anthocyanin content in response to water stress could have multiple benefits from a breeding perspective, aiming at enhancing the drought tolerance of the crop and the antioxidant properties of the food product. In lettuce, the great majority of transcriptomic studies related to anthocyanins are focused on the differences between green and red varieties (Moreno-Escamilla et al., 2020; Su et al., 2020). RNA-seq has also been used to study different abiotic stresses in this crop, e.g., high and low temperatures (Park et al., 2020; Chen et al., 2022a), the presence of heavy metals (Xiong et al., 2021), and even drought (Koyama et al., 2021). However, the specific effect of environmental factors on lettuce anthocyanin regulation has been scarcely studied, except in the case of different light conditions (Zhang et al., 2018; Wada et al., 2022).

Nowadays, RNA-seq is the most widely used technology for studying gene expression due to its many advantages. RNA-seq is a precise and sensitive technique that has also a wide range of detection and is highly accurate in terms of quantification (Wang et al., 2009). Despite being a powerful technique, some artefacts may be present in RNA-seq data (Everaert et al., 2017). Therefore, their validation with an independent technique like real-time quantitative PCR (qPCR) is advisable and even necessary when genes are small, have few exons or low levels of expression (Everaert et al., 2017).

Once differentially expressed genes (DEGs) have been identified, the study of polymorphisms in their sequences might provide information about functional and structural effects that could explain the observed variation for the trait of interest. However, the elucidation of these effects through experimental approaches is usually time and labour consuming and, in many cases, leads to dead ends. That is why the development and use of computational prediction tools as a first approach have experienced a boom in the last few years as they are able to provide increasingly more accurate information to assess phenotypic effects (Yazar and Özbek, 2021).

Metabolite-mediated drought adaptation is an emerging subject that has revealed the importance of some primary metabolites, such as sugars, small peptides, and amino acids, among others, in plant response, either acting as signal factors or as protectors (Zhang et al., 2024). Less is known about the participation of secondary metabolites (e.g., anthocyanins) in plant response to drought, beyond their antioxidant activity like scavengers of reactive oxygen species (ROS) (Naing and Kim, 2021). In this work, we have carried out transcriptomic analyses via RNA-seq and real-time qPCR in a red lettuce variety and a wild relative species that experienced a raise in anthocyanin content as a response to drought stress (Medina-Lozano et al., 2024). In addition, *in silico* predictions of the effects of polymorphisms in DEGs could potentially explain the observed differences between the two species in anthocyanin content in plants subject to water stress. The genetic knowledge of this response is key to obtaining new lettuce varieties with both enhanced drought tolerance and health-promoting properties, at the same time that water resources destined to irrigation could be cut down.

## 2 Materials and methods

### 2.1 Plant material

Two different accessions of the genus *Lactuca* were included in this study: a commercial variety, the red-leaf lettuce ‘Romired’, and a wild relative species, *Lactuca homblei* De Wild. They were selected from a previous drought stress experiment in which two irrigation regimes, control (C, week 1: 1350 mL, weeks 2-3: 2100 mL/each) and deficit irrigation (DI, weeks 1-3: 0 mL), were tested in two consecutive years (Medina-Lozano et al., 2024). Three biological replicates for the two accessions in each of the two conditions (C and DI) from the experiment carried out in winter 2020/2021 were used to proceed with the transcriptomic studies.

### 2.2 RNA extraction and sequencing, data processing and DEG identification

Total RNA extraction from lyophilized samples coming from 12 samples (2 accessions x 2 irrigation regimes x 3 biological replicates) was performed using the NZY Total RNA Isolation kit (NZYtech Lda.-Genes and Enzymes, Lisbon, Portugal) as described before (Medina-Lozano et al., 2023). RNA was treated with DNase using the TURBO DNA-free™ kit (Invitrogen, Waltham, MA, USA), following the manufacturers’ instructions. RNA quantity and purity were assessed with the Eukaryotic Total RNA Nanobioanalyzer Assay in a 2100 Bioanalyzer (Agilent Technologies, Santa Clara, CA, USA).

The obtained RNA samples from ‘Romired’ and *L. homblei* were used to perform the RNA-seq. They were processed to build a total of 12 strand-specific cDNA libraries. Sequencing of the libraries was performed in both directions with a NovaSeq 6000 S1 instrument (Illumina, San Diego, CA, USA) using the TruSeq Stranded mRNA protocol (Illumina) to obtain between 36 and 111 strand-specific pair-end reads of 100 base pair (bp) lengths per sample. Sequencing was carried out at the National Centre for Genomic Regulation (CNAG-CRG, Barcelona, Spain).

Sequences were analysed using the Galaxy tool (The Galaxy Community, 2022). Adapter sequences were removed by processing the reads from the 12 individual datasets using Trimmomatic (Galaxy version 0.38.1) (Bolger et al., 2014). RNA-seq data alignment to the lettuce reference genome *Lactuca sativa* ‘Salinas’ v8 (Reyes-Chin-Wo et al., 2017) was performed using HISAT2 (Galaxy version 2.2.1+galaxy0) (Kim et al., 2015), with a maximum intron length set at 20,000 bp. The Picard tools (http://broadinstitute.github.io/picard) MarkDuplicates (Galaxy version 2.18.2.2) and FixMateInformation (Galaxy version 2.18.2.1) were used to filter out the optical duplicates and to mate-pairs, respectively. featureCounts (Galaxy version 2.0.1+galaxy2) (Liao et al., 2013) was used to generate read counts using the gene annotation available in the literature (Reyes-Chin-Wo et al., 2017).

Analysis of differential gene expression between treatments (C and DI) within each of the two accessions was conducted using edgeR (Galaxy version 3.36.0+galaxy0) (Robinson et al., 2009). Genes were considered to be differentially expressed when values of |log_2_(FC, fold change)|>1 and FDR (False Discovery Rate)<0.05 (adjusted *p*-value via the Benjamini-Hochberg method).

### 2.3 Structural and functional analysis of the DEGs

Venn diagrams were performed with DEG datasets using the R stats package VennDiagram (https://CRAN.R-project.org/package=VennDiagram). GO (Gene Ontology) enrichment analyses were carried out using the tool GOEnrichment from Galaxy platform (Galaxy version 2.0.1) (Faria, 2017), with *p*-value cut-off < 0.05 and using Benjamini-Hochberg multiple test correction. Enriched GO terms involving three categories, biological processes, cellular components, and molecular functions, were evaluated. GO terms of the DEGs were obtained from predicted data using information available in the literature (Reyes-Chin-Wo et al., 2017). Heatmaps were constructed using gplots (https://CRAN.R-project.org/package=gplots) and ggplot2 (Wickham, 2009) R stats packages.

### 2.4 Selection of DEGs

The selection of genes for expression data validation was based on different criteria. First, genes were filtered out for values of |log_2_(FC)|>4 and FDR<0.05 (substantial increase or decrease in expression levels). Among them, those exhibiting high and significant correlation (both positive and negative) with, first, anthocyanin content and, second, drought stress treatment, were selected. Finally, gene functions related to both anthocyanin content and/or response to different stresses were also taken into account for the selection of a total of 19 DEGs.

Correlations between gene expression and both anthocyanin content and treatments were established through weighted gene co-expression network analysis (WGCNA), which was conducted using the R stats package WGCNA (Langfelder and Horvath, 2008). Normalised RNA-seq data of all genes were used for the WGCNA, except for those with a very low expression among the no DEGs (i.e., less than 5 reads per sample in the three biological samples of each group (C and DI)). Data from both species, *L. sativa* (cultivated lettuce ‘Romired’) and *L. homblei*, were analysed separately.

### 2.5 DEG validation using real-time qPCR

Total RNA was extracted from each of the 12 samples described above. Subsequently, mRNA was purified using the Dynabeads mRNA DIRECT™ kit (Invitrogen) and cDNA was synthesized using the NZY M-MuLV First-Strand cDNA Synthesis, separate oligos kit (NZYTech) as described before (Medina-Lozano et al., 2023).

Specific pairs of primers for each of the 19 selected DEGs (**Supplementary Table S1**) were designed using OLIGO software version 6.45 (Cascade, CO, USA) from a consensus sequence of the two species under study, *L. sativa* and *L. homblei*, excluding any ambiguity in the sequences. Real-time qPCR reactions were performed on a StepOnePlus™ System (Applied Biosystems, Waltham, MA, USA) with two technical replicates per each of the three biological replicates. Each reaction contained 1 µL of 1:5 diluted cDNA, 0.40 µM of forward and reverse primers (Integrated DNA Technologies, IDT, Coralville, Iowa, USA), and 1x NZYSupreme qPCR Green Master Mix, ROX plus (NZYTech) in a final volume of 11 µL. The amplification conditions were: 2 min at 95°C, 40 cycles of 5 s at 95°C, 15 s at 52-66°C (**Supplementary Table S1**) and 30 s at 72°C, followed by the melting curve analysis that ranged from 72°C to 95°C with 0.3°C increment per cycle to verify that a single product was amplified. Non-template controls were included to ensure that contamination with genomic DNA had not occurred.

*TRXL3-3* was used as reference gene to normalise qPCR data (Medina-Lozano et al., 2023). Relative expression levels were obtained using the Pfaffl method (Pfaffl, 2001) with some modifications: arithmetic instead of geometric mean was calculated due to the presence of zero values in the raw data (either genes completely shut down as a consequence of the DI or the other way round, unexpressed genes in C conditions that were activated with the DI). This explains values different from 1 in C samples and why they have been represented separately from the DI data in qPCR results.

Student *t*-test was used to assess whether the differences between the means from the qPCR expression data of samples under C and DI conditions were statistically significant. Data transformations (1/(1+x)^2^ or 1/√(x+1)) were applied when needed to achieve a normal distribution. Alternatively, Wilcoxon test was used with non-normally distributed data. Statistical analyses were conducted using the software JMP v5.1.2 for Windows (SAS Institute Inc. Cary, NC).

### 2.6 Polymorphism search, annotation, and effect prediction in the DEGs

Detection of polymorphisms was carried out using the sequences of the 12 samples aligned to the lettuce reference genome (Reyes-Chin-Wo et al., 2017) and processed as explained in subsection 2.2. Firstly, variant calling was performed using FreeBayes package (Galaxy Version 1.3.6+galaxy0) (Tange, 2011; Garrison and Marth, 2012) from Galaxy platform. Then, VCFfilter (Galaxy Version 1.0.0_rc3+galaxy3) (Garrison, 2015) was used to remove polymorphisms with a total read depth at the locus < 10, QUAL < 20, and number of alternative alleles in called genotypes > 0. In addition, those polymorphic sites exhibiting different genotypes among the total number of samples within accessions and/or more than two different genotypes in comparison with the reference genome in more than 70% of the cases, were filtered out with Excel. Any possible ambiguous polymorphism was also eliminated.

The effect of each polymorphism was annotated and predicted using the SnpEff eff tool (Galaxy Version 4.3+T.galaxy1) and a snpEff database created using the SnpEff build tool (Galaxy Version 4.3+T.galaxy4) (Cingolani et al., 2012) from the annotation dataset and the FASTA file of *L. sativa* ‘Salinas’ v8 (Reyes-Chin-Wo et al., 2017).

## 3 Results

### 3.1 Transcriptome analysis

To investigate the involvement of anthocyanins at molecular level in the response mechanism to drought stress of *Lactuca* spp., a transcriptomic analysis via RNA-seq was performed using plants belonging to the CWR *L. homblei* and to the red commercial lettuce variety ‘Romired’ coming from a previous experiment carried out in winter 2020/2021 (Medina-Lozano et al., 2024). Samples of both accessions showed an accumulation of anthocyanins under DI in comparison to C conditions, though the differences only resulted statistically significant in the case of the wild species *L. homblei* (Medina-Lozano et al., 2024). In particular, three different anthocyanins were identified: cyanidin 3-*O*-(6’-*O*-malonylglucoside) was the predominant one and was detected in both accessions and treatments; peonidin 3-*O*-glucoside appeared under both treatments in the commercial variety, but only under DI in the CWR; and cyanidin 3-(6’’-acetylglucoside), exclusively identified under DI conditions in the commercial variety.

After processing the data from *L. homblei*, the clean reads ranged from 40.76 to 51.75 Gb and the percentage of uniquely mapped sequences to the reference genome ranged from 32.39% to 37.40%. In the case of ‘Romired’, the clean reads ranged from 35.43 to 110.62 Gb and the uniquely mapped sequences from 81.04% to 84.81% (**Table 1**). RNA-seq data from both accessions were aligned to the lettuce reference genome *L. sativa* ‘Salinas’ v8 (Reyes-Chin-Wo et al., 2017). However, *L. homblei* belongs to the tertiary lettuce gene pool (PGR (Plant Genetic Resources) Lettuce, https://www.pgrportal.nl/en/lettuce-geneticresources-portal.htm), so it is quite distant from *L. sativa*, what might explain its lower values in terms of uniquely mapped sequences.

**Table 1:** Statistical summary of RNA-sequencing data.

Group^a^	Sample	Raw reads	Clean reads	Mapped reads	Mapping rate (%)	GC (%)
*L. homblei* C	*L. homblei* 1	51,853,084	51,749,303	19,128,708	36.96	44.5
	*L. homblei* 2	46,166,391	46,074,390	17,230,542	37.40	45.5
	*L. homblei* 3	42,697,894	42,615,564	15,099,405	35.43	43.5
*L. homblei* DI	*L. homblei* 4	43,141,038	43,063,549	15,173,600	35.24	45.0
	*L. homblei* 5	40,836,180	40,756,877	13,200,520	32.39	45.0
	*L. homblei* 6	46,624,421	46,532,339	16,185,998	34.78	44.0
‘Romired’ C	‘Romired’ 1	35,523,451	35,428,117	29,244,585	82.55	41.5
	‘Romired’ 2	101,124,691	100,936,321	85,092,426	84.30	44.5
	‘Romired’ 3	110,827,310	110,624,887	93,466,553	84.49	45.0
‘Romired’ DI	‘Romired’ 4	94,626,862	94,435,273	80,092,100	84.81	45.0
	‘Romired’ 5	52,365,850	52,260,702	42,353,540	81.04	45.0
	‘Romired’ 6	41,007,770	40,932,006	34,429,220	84.11	45.0

a C, control; DI, deficit irrigation.

### 3.2 Identification and analysis of DEGs under drought stress conditions

**Figure 1:**
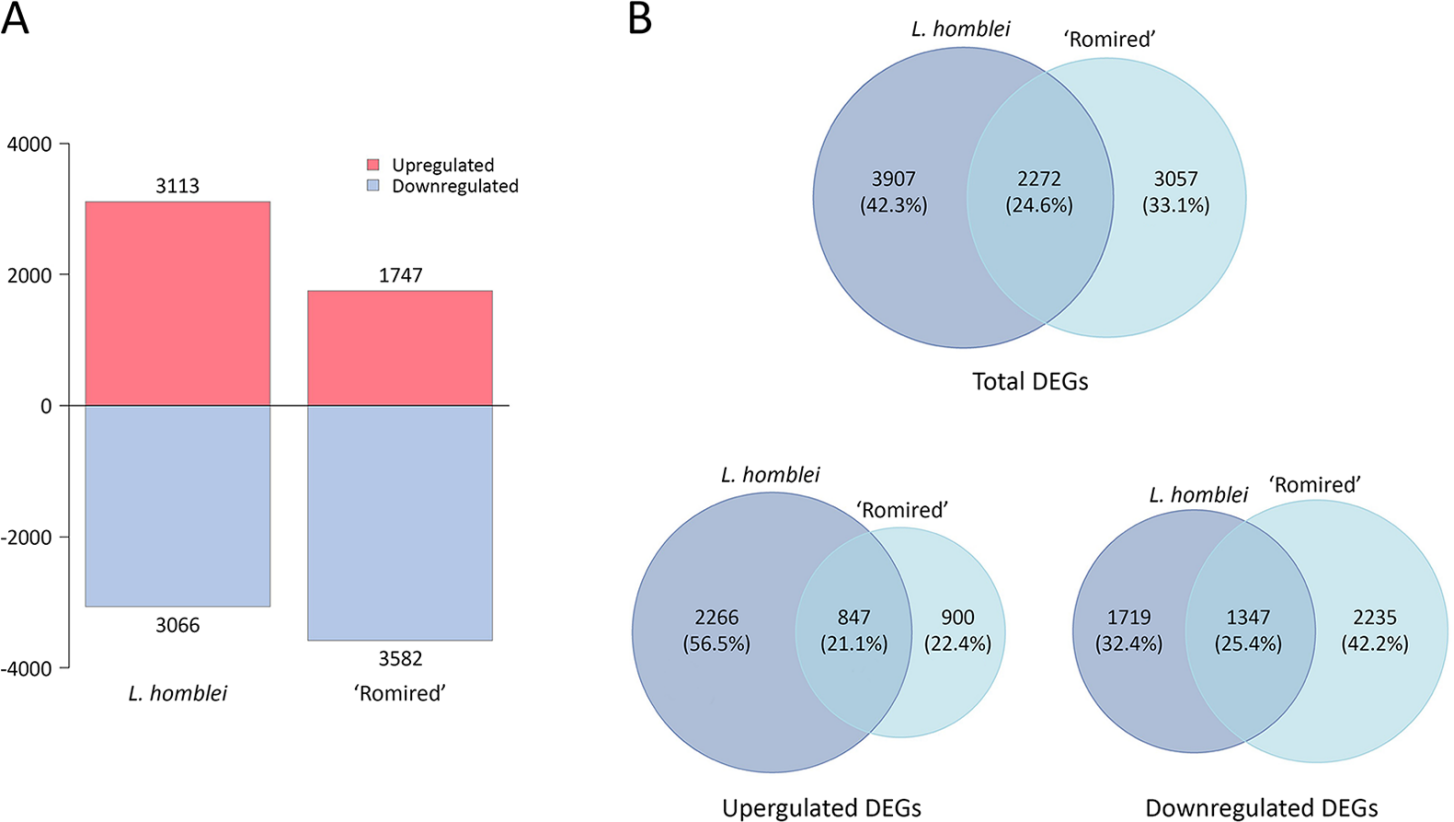
Differentially expressed genes (DEGs) in *Lactuca* spp. in response to drought stress. **(A)** Number of upregulated and downregulated DEGs in *L. homblei* and ‘Romired’. **(B)** Venn diagrams showing the common and exclusive number of genes within the total, upregulated, and downregulated DEGs.

A total of 6,179 DEGs were identified when *L. homblei* plants under C and DI treatments were compared (3,113 upregulated and 3,066 downregulated genes), whereas a total of 5,329 DEGs were obtained in ‘Romired’ plants for the same treatments (1,747 upregulated and 3,582 downregulated genes) (**Figure 1A**). A total of 2,272 DEGs were common to both accessions: 847 genes were upregulated, 1,347 downregulated, and 78 exhibited an opposite behaviour in the two accessions (**Figure 1B**). Attending to the differences, the CWR showed a total number of DEGs higher than the cultivated species (42.3% of exclusive DEGs in *L. homblei* vs. 33.1% in ‘Romired’). The same happened in the case of the upregulated genes, where the disparity was the largest, a 56.5% of the DEGs was exclusively upregulated in *L. homblei*, which was more than twice the upregulated DEGs only in ‘Romired’ (22.4%). In the case of the downregulated genes, we observed the opposite, the number was higher in the cultivated species than in the wild relative (42.2% vs. 32.4%, respectively) (**Figure 1B**).

**Figure 2:**
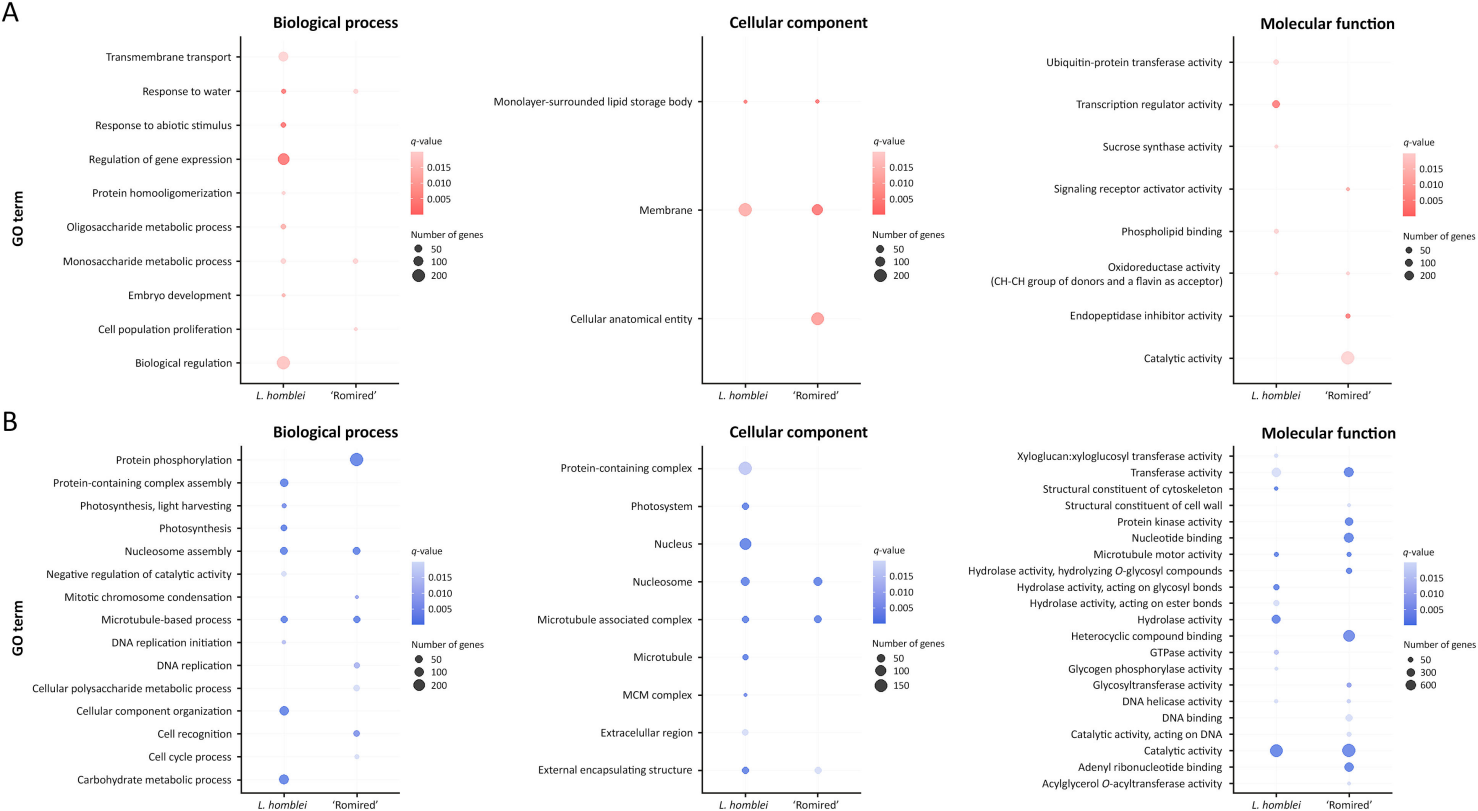
Enriched GO terms of *L. homblei* and ‘Romired’ within the **(A)** upregulated and **(B)** downregulated differentially expressed genes (DEGs) identified in a drought stress experiment for the three main GO categories: biological process, cellular component, and molecular function.

To deeply explore the DEG functions in the drought response of *Lactuca* spp., analyses of GO enrichment were conducted using the GO annotations found in Reyes-Chin-Wo et al. (2017). The three main GO categories, biological processes, cellular components and molecular functions, were studied within the upregulated and downregulated genes (**Figure 2**). Within the upregulated genes, the number of enriched GO terms in biological processes was higher in *L. homblei* than in ‘Romired’ (**Figure 2A**). In particular, an important number of *L. homblei* DEGs belonged to significantly enriched GOs that were involved in transmembrane transport and different metabolic processes, though the most significantly upregulated DEGs were those in enriched GOs related to gene expression regulation and response to abiotic stimulus, water included. The response to water resulted to be also among the enriched GO terms in ‘Romired’, but the number of genes, and especially the significance level, were lower than in the CWR *L. homblei*. In the case of cellular components (**Figure 2A**), a similar number of genes were part of the GO terms membranes and lipid storage bodies in both species, with a higher significance in ‘Romired’. In addition, the GO term cellular anatomical entity was enriched exclusively in the cultivated accession. Finally, for the molecular function category (**Figure 2A**), the most significantly enriched term was the transcription regulator activity in the CWR *L. homblei*, and the endopeptidase inhibitor activity in the commercial lettuce ‘Romired’. Interestingly, anthocyanins (among other flavins) could be involved in the oxidoreductase activity in which a flavin group acts as acceptor, being actually the only enriched GO common to both species.

Within the downregulated DEGs of biological processes (**Figure 2B**), genes involved in the carbohydrate metabolism were the most represented in *L. homblei*, while in ‘Romired’ were those implied in protein phosphorylation, that in fact, appeared only in this accession. However, several enriched processes, as well as their significance levels, were common or similar in both species, such as those related to cellular division and multiplication (DNA replication, nucleosome assembly, and microtubule-based processes). In the cellular component category (**Figure 2B**), the enriched GO terms found in ‘Romired’ appeared also enriched in *L. homblei*: nucleosome, microtubule associated complex, and external encapsulating structure, with similar significance and number of genes, except for the external encapsulating structure that resulted more significant in *L. homblei*. In fact, many more GO terms were enriched in *L. homblei*, with the nucleus and the protein-containing complex being the most represented ones. On the contrary, we found many more downregulated DEGs with enriched GO, as well as more terms and with a higher significance, in the cultivated (‘Romired’) than in the wild species (*L. homblei*) in the molecular function category (**Figure 2B**).

Some DEGs were assigned to more than one GO term, either because a gene can participate in different biological processes and molecular functions and be part of different cellular components or because GO is loosely hierarchical, with genes belonging to both ‘parent’ and ‘child’ terms. Thus, counting genes only once within each category, we obtained that the number of DEGs with enriched GO terms was very similar between *L. homblei* and ‘Romired’ within the upregulated genes (746 vs. 794, respectively), while it was lower in the CWR *L. homblei* than in the commercial variety ‘Romired’ within the downregulated ones (1,591 vs. 1978, respectively). Even so, the percentages of common DEGs in the two species was considerably lower in the case of upregulated genes than in downregulated: 3.23%, 18.25%, and 1.14% vs. 12.81%, 22.98%, and 23.00% in biological processes, cellular components and molecular functions, respectively.

**Figure 3:**
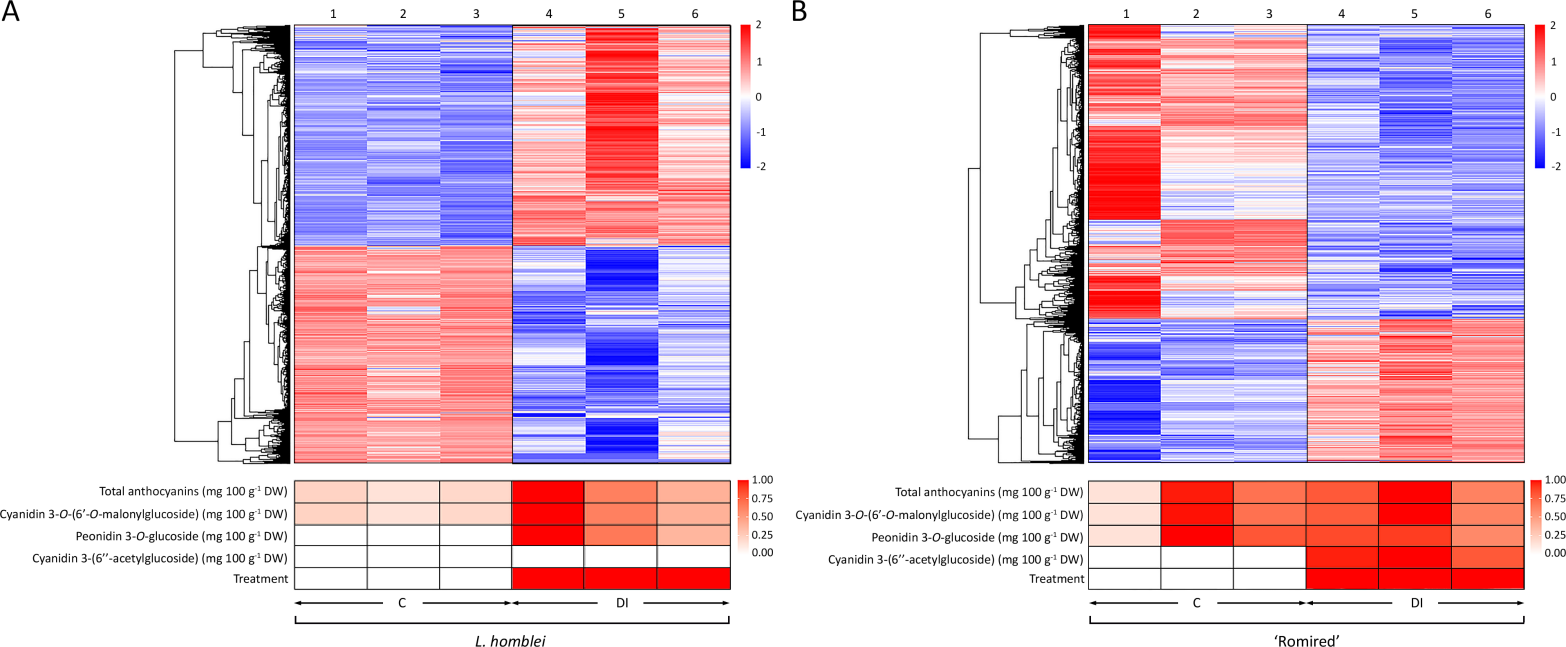
Heatmap representation of hierarchical analysis of the expression data from the differentially expressed genes (DEGs), as well as of total and individual anthocyanin content and treatment in **(A)** *L. homblei* and **(B)** ‘Romired’ under control (C) and deficit irrigation (DI) conditions. Numbers 1-3 and 4-6 show the biological replicates under C and DI, respectively. Phenotypic heatmaps represent scaled data from 0.2 to 1 within each compound, except for minor anthocyanins (peonidin 3-*O*-glucoside in *L. homblei* and cyanidin 3-(6’’-acetylglucoside) in ‘Romired’) where data, as well as treatments, were scaled from 0 to 1.

Two heatmaps constructed using normalised expression data of DEGs in *L. homblei* and ‘Romired’ confirmed the effect of the drought stress treatment in *Lactuca* spp. plants in terms of gene regulation (**Figure 3**). A hierarchical clustering conducted with all the DEGs allowed us to identify two separate groups in the two accessions, as expected, the upregulated and the downregulated ones. This clustering also divided clearly the two conditions (C and DI) in both species, what was even more evident for *L. homblei* (**Figure 3A**). In addition, both heatmaps showed again that the number of upregulated genes under water deficit was clearly higher in the CWR *L. homblei* (**Figure 3A**), whereas those downregulated were more numerous in the commercial variety ‘Romired’ (**Figure 3B**). These results show that the wild species was activating more mechanisms in response to drought stress. **Figure 3** also shows two heatmaps constructed using the data of the anthocyanin content variation as a consequence of the drought stress for *L. homblei* and ‘Romired’ (Medina-Lozano et al., 2024), and the data of the two treatments themselves (C and DI). Content of all detected anthocyanins was higher under DI treatment than in C conditions in both *Lactuca* spp. Similar to what happened with the upregulated genes, the accumulation of anthocyanins in response to water stress was higher (and only significant) in *L. homblei* (**Figure 3A**). Actually, in *L. homblei* all the DI replicates showed a higher content than the C replicates. This was especially remarkable in the case of peonidin 3-*O*-glucoside, which was present under DI conditions and in the 3 biological replicates, but not under C conditions (**Figure 3A**). However, differences were not so clear (and not significant) between DI and C replicates in ‘Romired’, despite mean anthocyanin content being higher in DI than in C conditions, as commented above (**Figure 3B**). Even though, one of the anthocyanins was also identified exclusively under DI conditions in ‘Romired’, as observed in *L. homblei*, but in this case, it was cyanidin 3-(6’’-acetylglucoside) (**Figure 3B**).

**Figure 4:**
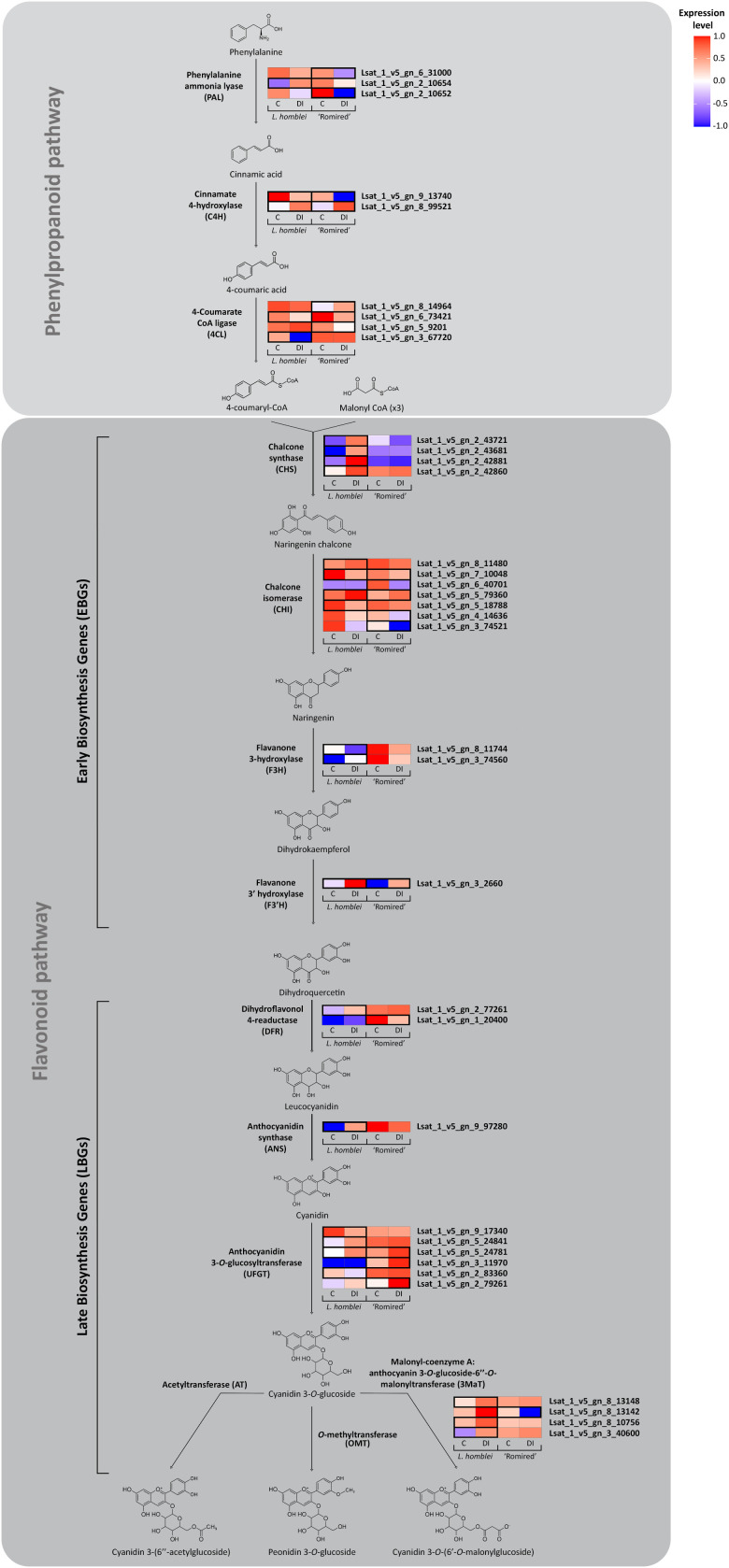
Simplified pathway for the anthocyanin biosynthesis of differentially expressed genes (DEGs) detected in *L. homblei* and/or ‘Romired’ under control (C) and deficit irrigation (DI) conditions. Heatmaps represent the expression data scaled from -1 to 1 for each isoform of the DEGs (same names as the enzymes that catalyse each step) identified in the RNA-seq analysis. Black boxes indicate the accession in which the genes were differentially expressed (|log_2_(FC)|>1.06).

Nevertheless, the molecular mechanisms underlying the anthocyanin accumulation as a consequence of water deficiency have been barely studied in lettuce, unlike in other crops like grapevine (Castellarin et al., 2007) or canola (Chen et al., 2022b). To gain a more comprehensive understanding of the process in *Lactuca* spp., we mapped the expression profiles of the DEGs identified in the drought experiment which participate in the biosynthesis pathway of the detected anthocyanins (the general phenylpropanoid pathway and the flavonoid pathway, this last one leading specifically to the anthocyanin biosynthesis) (**Figure 4**). We found a total of 36 DEGs involved in the pathway in either *L. homblei*, ‘Romired’ or both. Different expression profiles were observed between both species. Our results confirmed that, in these routes, more DEGs were activated in the CWR *L. homblei* than in the commercial variety ‘Romired’, 17 vs. 7 upregulated genes, respectively, what was concordant with the higher accumulation of anthocyanins in the wild relative (Medina-Lozano et al., 2024). Not all the isoforms of the genes coding for the enzymes catalysing each step were upregulated under DI. The activation happened mainly in the first steps of the pathway, that is, in the early biosynthesis genes (EBGs), especially at the beginning of anthocyanin-specific route (flavonoid pathway). This becomes glaringly obvious in the first step which is catalysed by the chalcone synthase (CHS), whose gene isoforms are all strongly and significantly upregulated only in *L. homblei* (triggering of the anthocyanin synthesis in the wild species). This pattern is not so obvious in the preceding genes from the general phenylpropanoid pathway as they participate in the biosynthesis of many other compounds apart from anthocyanins. Furthermore, the late biosynthesis genes (LBGs) were mostly upregulated in the CWR *L. homblei* but not in all the isoforms as observed in *CHS* (EBG), except in the last step which leads to the synthesis of the specific major anthocyanin (cyanidin 3-*O*-(6’-*O*-malonylglucoside)) where most of the genes coding for the isoforms were significantly activated in *L. homblei*. The final steps to produce the two minor anthocyanins, cyanidin 3-(6’’-acetylglucoside) and peonidin 3-*O*-glucoside, are not clearly described in the literature. They might be catalysed by some acetyltransferases and *O*-methyltransferases, respectively, as suggested by Ino and Yamaguchi (1993) and Hugueney et al. (2009), respectively. It is possible that the genes coding for these enzymes were activated under drought as those anthocyanins were detected in ‘Romired’ and *L. homblei*, respectively, only under stress conditions though they have not been characterised in *L. sativa* yet.

### 3.3 Selection of candidate genes among the DEGs

Selection of anthocyanin-related genes potentially involved in the response to drought stress was based on different criteria. First, we searched for important changes in the expression levels. Second, we selected DEGs with high (positive and negative) and significant values of correlation with both anthocyanin content and drought stress treatment, obtained through a WGCNA. WGCNA allows to identify genes correlated with certain traits (anthocyanins and irrigation treatment in our case) to reveal putative genes with particular interest (Horvath and Dong, 2008). Lastly, we paid attention to gene function, so that DEGs were related to stress and/or anthocyanin content. Finally, 19 genes were selected for validation through real-time qPCR. Remarkably, genes meeting all these criteria resulted to be differentially expressed exclusively in *L. homblei*.

*L. homblei* |log_2_(FC)| values between C and DI ranged from 4.03 to 6.23 (**Table 2**). Both up- and downregulated genes were included in the selection. The higher accumulation of anthocyanins and/or the activation of stress response may result either from the upregulation of activators or from the downregulation of repressors. This was also observed in a previous study that characterised four genes related to anthocyanin content in lettuce (Su et al., 2020). From the WGCNA results, we obtained absolute correlation values with anthocyanins ranging from 0.81 to 0.93, and with treatment, from 0.86 to 0.99 (**Table 2**). Both positive and negative correlations were also considered here. By contrast, in the case of ‘Romired’, we found 5 out of the 19 genes showing a significant correlation with the anthocyanin content, but none of them exhibited a significant change of expression level nor a significant correlation with the stress treatment (data not shown).

**Table 2:** Gene product, regulation and correlation with total anthocyanin content and treatment of the 19 differentially expressed genes (DEGs) selected.

					Total anthocyanins		Irrigation treatment	
Gene ID	Gene product	Regulation	Log_2_(FC)^a^	FDR^b^	Correlation coefficient	*p* value	Correlation coefficient	*p* value
Lsat_1_v5_gn_1_21441	Subtilisin-like protease SBT3	Downregulated	-4.03	3.33E-04	-0.81	0.05	-0.96	2.85E-03
Lsat_1_v5_gn_1_50480	Haloacid dehalogenase (HAD)-like hydrolase superfamily protein	Downregulated	-4.28	3.33E-04	-0.93	0.01	-0.86	0.03
Lsat_1_v5_gn_1_109200	DNA damage-repair/toleration protein DRT100	Downregulated	-4.44	2.27E-03	-0.82	0.05	-0.91	0.01
Lsat_1_v5_gn_1_127541	14 kDa proline-rich protein DC2.15	Downregulated	-5.14	2.53E-03	-0.93	0.01	-0.91	0.01
Lsat_1_v5_gn_2_15680	GDSL esterase/lipase	Downregulated	-4.81	0.04	-0.93	0.01	-0.89	0.02
Lsat_1_v5_gn_2_43400	Probable pectate lyase 8	Downregulated	-5.52	2.25E-03	-0.87	0.02	-0.99	1.75E-04
Lsat_1_v5_gn_2_47181	Protein ECERIFERUM 26	Downregulated	-5.16	0.03	-0.92	0.01	-0.91	0.01
Lsat_1_v5_gn_2_90361	Gibberellin-regulated protein 6	Downregulated	-4.29	0.03	-0.93	0.01	-0.95	3.94E-03
Lsat_1_v5_gn_2_116640	Heat shock cognate 70 kDa protein 2	Upregulated	5.53	6.34E-06	0.84	0.03	0.97	1.06E-03
Lsat_1_v5_gn_3_1101	Zinc finger protein ZAT1	Downregulated	-4.75	1.31E-03	-0.90	0.01	-0.98	5.92E-04
Lsat_1_v5_gn_3_20640	PRA1 family protein E	Upregulated	4.18	1.18E-03	0.82	0.04	0.98	8.11E-04
Lsat_1_v5_gn_5_7401	NAC transcription factor 56	Upregulated	4.22	7.39E-04	0.82	0.05	0.94	0.01
Lsat_1_v5_gn_5_10141	Protein MHF1 homolog	Downregulated	-4.66	0.04	-0.91	0.01	-0.86	0.03
Lsat_1_v5_gn_5_26000	B-box zinc finger protein 21-like	Upregulated	4.34	2.38E-05	0.82	0.05	0.93	0.01
Lsat_1_v5_gn_6_67540	Type I inositol polyphosphate 5-phosphatase 2	Downregulated	-4.32	7.53E-07	-0.85	0.03	-0.93	0.01
Lsat_1_v5_gn_7_92980	Ribonuclease III-like protein RTL3	Downregulated	-6.23	3.97E-03	-0.92	0.01	-0.93	0.01
Lsat_1_v5_gn_8_157561	Transcription factor MYC/MYB N-terminal domain-containing protein	Downregulated	-4.46	4.60E-04	-0.84	0.04	-0.94	4.91E-03
Lsat_1_v5_gn_8_165301	Phospholipase A1 phospholipid-inositol phosphatase PLIP2	Upregulated	4.28	4.73E-05	0.84	0.04	0.96	1.94E-03
Lsat_1_v5_gn_9_80621	Amino acid permease 6	Upregulated	4.17	2.91E-03	0.82	0.04	0.96	2.81E-03

a FC, fold change.

b FDR, False Discovery Rate.

### 3.4 Validations of candidate genes by qPCR

**Figure 5:**
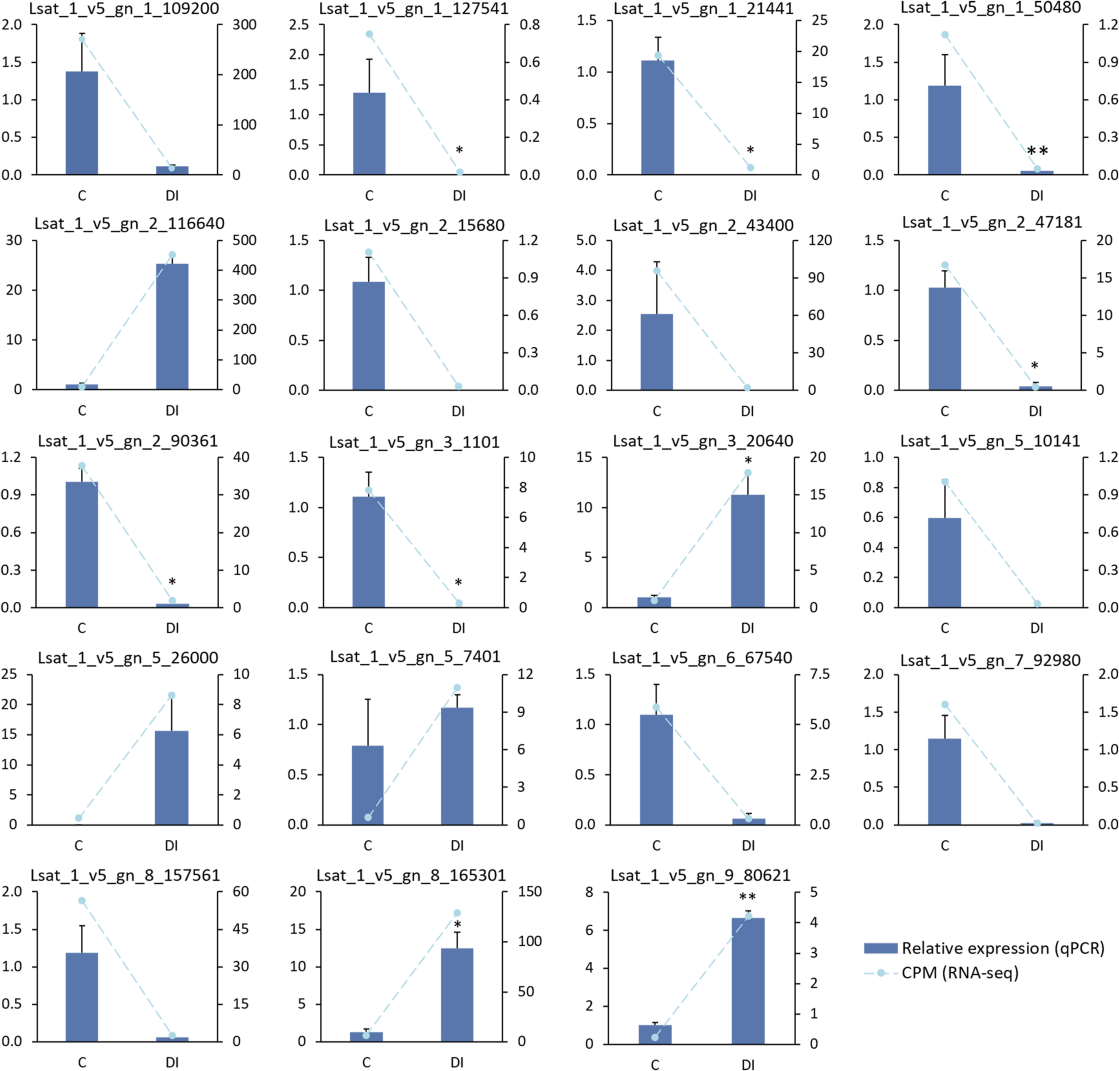
Expression data obtained by qPCR (relative expression) and by RNA-seq (CPM, counts per million) of 19 selected genes in the wild species *L. homblei* under control (C) and deficit irrigation (DI) conditions. Bars represent standard error of the total (n=3). *p<0.05, **p<0.01. Transformations were applied to achieve normal distribution in qPCR data in the following cases: 1/(1+x)^2^ to Lsat_1_v5_gn_1_109200, Lsat_1_v5_gn_1_127541, Lsat_1_v5_gn_1_50480, Lsat_1_v5_gn_2_116640, Lsat_1_v5_gn_2_43400, and Lsat_1_v5_gn_3_20640; and 1/√(x+1) to Lsat_1_v5_gn_1_21441. Wilcoxon test was used with non-normally distributed qPCR data of Lsat_1_v5_gn_5_10141 and Lsat_1_v5_gn_5_26000.

The expression data of the 19 selected genes obtained from the RNA-seq analysis were validated by real-time qPCR. In *L. homblei*, the 13 downregulated and the 6 upregulated genes according to the RNA-seq analysis showed concordant expression profiles with the qPCR results (**Figure 5**). Significant, and very significant differences were observed between C and DI treatments for seven and two genes, respectively, according to qPCR data (**Figure 5**). In the case of ‘Romired’, the selected genes did not show any differential expression in the RNA-seq analysis, as mentioned above, nor by qPCR. Even so, the expression of 15 out of the 19 genes followed the same profile using the two different techniques (**Supplementary Figure S1**). Therefore, we were able to confirm the reliability of the results from the RNA-seq analysis.

**Figure 6:**
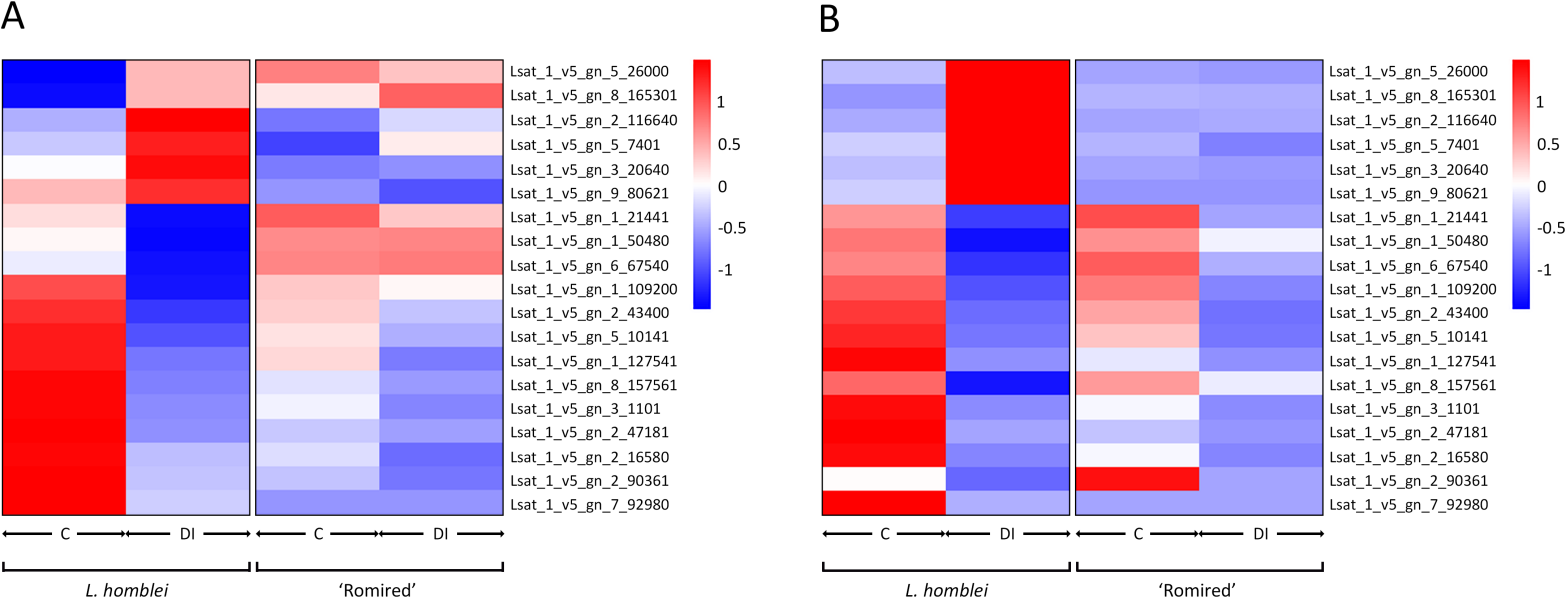
Heatmap representation of hierarchical analysis of the mean expression data (n=3) of 19 selected genes in *L. homblei* and ‘Romired’ under control (C) and deficit irrigation (DI) conditions according to **(A)** RNA-seq and **(B)** real-time qPCR analyses.

The expression profiles of the selected DEGs obtained with both techniques (RNA-seq and qPCR) were also analysed by hierarchical clustering both using the mean values (**Figure 6**) and all data (**Supplementary Figure S2**). According to the RNA-seq data, two clearly differentiated expression patterns could be observed for *L. homblei*: the expression levels were noticeably lower in C than in DI conditions in the case of upregulated genes, and vice versa in the case of downregulated genes (**Figure 6A**; **Supplementary Figure S2A**). In contrast, for ‘Romired’ the differences in expression were not clear in most of the selected genes (**Figure 6A**; **Supplementary Figure S2A**), as was expected since they did not result to be differentially expressed in the RNA-seq analysis. Comparable patterns, especially for *L. homblei* samples, were observed when qPCR data were represented (**Figure 6B**; **Supplementary Figure S2B**).

### 3.5 Putative function of validated candidate genes

Turning the attention to gene function, it could be confirmed that those 19 DEGs with large changes in expression levels and high correlations with treatment and anthocyanins were indeed related to stress responses and/or to anthocyanin content (**Table 3**). Specifically, most gene products of the selected DEGs have been described to participate in the response to one or more types of stresses. Several of the DEGs are involved in the response to biotic stresses, like resistance to bacteria (Lsat_1_v5_gn_1_21441 (Ramírez et al., 2013)), virus (Lsat_1_v5_gn_3_1101 (Tsitsekian et al., 2023)), or fungi (Lsat_1_v5_gn_3_20640 (Wu et al., 2022)), but most have been described to act in abiotic stress responses. In particular, genes related to water deficit and/or the stress-responsive hormone ABA (abscisic acid) stood out, such as Lsat_1_v5_gn_2_116640 (Clément et al., 2011), Lsat_1_v5_gn_2_43400 (Palusa et al., 2007), and Lsat_1_v5_gn_3_20640 (Tahmasebi et al., 2019). Furthermore, not only genes reported to be generally activated under water stress conditions were included in the selection, but also some described as negative regulators, which were in fact downregulated (inhibition of suppressors) in our samples subject to the drought treatment (**Tables 2**, **3**), like Lsat_1_v5_gn_1_50480 (Lee et al., 2022), Lsat_1_v5_gn_2_90361 (Qu et al., 2016), and Lsat_1_v5_gn_6_67540 (Na and Metzger, 2020). Some of the selected DEGs have been related to other abiotic stresses such as salt (Lsat_1_v5_gn_1_127541 (Bhattarai et al., 2021) and Lsat_1_v5_gn_3_1101 (He et al., 2020)), heat (Lsat_1_v5_gn_2_47181 (Zhang et al., 2022)), and nutrient deficiency (Lsat_1_v5_gn_9_80621 (Zhou et al., 2021)), which makes sense as especially salt and heat stresses often occur simultaneously with drought.

**Table 3:** Putative function of the 19 candidate differentially expressed genes (DEGs) in *L. homblei*.

Gene ID	Gene product	Putative function	Reference
Lsat_1_v5_gn_1_21441	Subtilisin-like protease SBT3	Plant immune priming in systemic induced resistance establishment	Ramírez et al., 2013
Lsat_1_v5_gn_1_50480	Haloacid dehalogenase (HAD)-like hydrolase superfamily protein	Repression of ABA-response and ABA-mediated drought tolerance	Lee et al., 2022
Lsat_1_v5_gn_1_109200	DNA damage-repair/toleration protein DRT100	Repair and toleration of UV-B-induced DNA damage	Fujimori et al., 2014
Lsat_1_v5_gn_1_127541	14 kDa proline-rich protein DC2.15	Cell wall modification and organization	Bhattarai et al., 2021
Lsat_1_v5_gn_2_15680	GDSL esterase/lipase	Flavonoid accumulation and lipid reduction under drought stress	Li et al., 2020
Lsat_1_v5_gn_2_43400	Probable pectate lyase 8	Response to stimulus through cell wall modification	Palusa et al., 2007
Lsat_1_v5_gn_2_47181	Protein ECERIFERUM 26	Dehydration tolerance under heat stress	Zhang et al., 2022
Lsat_1_v5_gn_2_90361	Gibberellin-regulated protein 6	ABA-repressible peptide hormone precursor	Qu et al., 2016
Lsat_1_v5_gn_2_116640	Heat shock cognate 70 kDa protein 2	ABA-induced stomatal closure	Clément et al., 2011
Lsat_1_v5_gn_3_1101	Zinc finger protein ZAT1	Putative transcription factor that acts in the response to abiotic and biotic stresses	He et al., 2020; Tsitsekian et al., 2023
Lsat_1_v5_gn_3_20640	PRA1 family protein E	Protein transporter involved in abiotic and biotic stress responses	Tahmasebi et al., 2019; Wu et al., 2022
Lsat_1_v5_gn_5_7401	NAC transcription factor 56	Transcription factor that induces anthocyanin accumulation	Wei et al., 2020
Lsat_1_v5_gn_5_10141	Protein MHF1 homolog	DNA repair and homologous recombination	Dangel et al., 2014
Lsat_1_v5_gn_5_26000	B-box zinc finger protein 21-like	Positive transcriptional regulator of light-induced anthocyanin accumulation	Zhang et al., 2021
Lsat_1_v5_gn_6_67540	Type I inositol polyphosphate 5-phosphatase 2	Putative repressor of water stress response	Na and Metzger, 2020
Lsat_1_v5_gn_7_92980	Ribonuclease III-like protein RTL3	Cleavage of double strand RNA	Comella et al., 2008
Lsat_1_v5_gn_8_157561	Transcription factor MYC/MYB N-terminal domain-containing protein	Putative transcriptional repressor of anthocyanin biosynthesis	Yan et al., 2021
Lsat_1_v5_gn_8_165301	Phospholipase A1 phospholipid-inositol phosphatase PLIP2	ABA-mediated abiotic stress responses and anthocyanin accumulation	Wang et al., 2018
Lsat_1_v5_gn_9_80621	Amino acid permease 6	AA transport under nutrient stresses	Zhou et al., 2021

Genes related to flavonoid (e.g., anthocyanins) accumulation were mostly upregulated in our samples (**Tables 2** and **3**). Two of those genes encode putative transcription factors (TFs): a zinc finger protein (Lsat_1_v5_gn_5_26000 (Zhang et al., 2021)) and a NAC TF (Lsat_1_v5_gn_5_7401 (Wei et al., 2020)), both having been described to induce anthocyanin-related genes. Among the DEGs identified in this work, other two have been previously described to cause the increase of flavonoid or anthocyanin content under abiotic stress. Specifically, Lsat_1_v5_gn_2_15680 was found to play important roles in flavonoid accumulation under drought stress in tea (Li et al., 2020), and Lsat_1_v5_gn_8_165301 was involved in the increase of anthocyanin content when overexpressed in *Arabidopsis thaliana* under ABA-mediated abiotic stress responses (Wang et al., 2018). Another selected gene (Lsat_1_v5_gn_8_157561) is not well characterised but could possibly be related to the anthocyanin content as it contains a MYB domain and, in plants, MYB TFs have been described as one of the major transcriptional regulators of anthocyanin pathway, both activators and repressors (Yan et al., 2021; Cao et al., 2024). This specific gene was downregulated in our samples when exposed to water stress, so it might be a transcriptional repressor of anthocyanin biosynthesis. Lsat_1_v5_gn_7_92980 encodes a ribonuclease III-like protein 3 (RTL3) that cleaves doble-stranded RNA (Comella et al., 2008) and might be also related to anthocyanin content. Proteins with ribonuclease III domains have been described to participate in the regulation of seed coat in soybean and fruit colour in peach through the production of siRNAs (small interfering RNAs) and the increase of transcription levels of genes implied in anthocyanin regulation (Zhu et al., 2012; Jia et al., 2020). Finally, two genes that encode proteins involved in DNA damage repair were also selected (Lsat_1_v5_gn_1_109200 (Fujimori et al., 2014), and Lsat_1_v5_gn_5_10141 (Dangel et al., 2014)). This is not surprising as the generation of ROS is a potential cause of DNA damage under drought stress and a fine-tuned regulation of DNA repair is required to tolerate it (Shim et al., 2018).

### 3.6 Polymorphisms in the DEGs

*In silico* search and prediction of polymorphisms were performed to get an overview of the variation in the sequences of the total number of DEGs detected and to explore more deeply the structural variation of the set of 19 selected DEGs.

A total of 235,600 polymorphisms were found in the whole set of DEGs (9,236) in *L. homblei* and ‘Romired’ compared to the reference genome (both shared and species-exclusive). Most polymorphisms were detected in *L. homblei*, as expected since it is a wild species that is very distant from the cultivated *L. sativa* used as reference. The predominant types of polymorphisms were SNPs (Single Nucleotide Polymorphisms) (89.22%), followed by MNPs (Multiple Nucleotide Polymorphisms) (9.97%), and, in a much smaller extent, by indels (insertions-deletions) (0.81%). The most abundant polymorphism effects were synonymous (63.20%) and missense (27.96%). We also identified intron (6.14%) and splice region (2.23%) variants, as well as others that were present in less than 0.1%, so they are not detailed here.

In the subset of 19 DEGs selected, a total of 404 polymorphisms with 408 predicted effects were identified in *L. homblei* (**Table 4**), in contrast to the 11 polymorphisms with 12 predicted effects found in those same 19 genes non-differentially expressed in ‘Romired’ (**Supplementary Table S2**). Considering only the 19 DEGs in *L. homblei*, the proportions of both polymorphism types and effects were almost the same than those found in the whole set of DEGs, 87.87% of polymorphisms were SNPs, 11.14% were MNPs, and 0.99% were indels. Once again, we found that the predominant predicted effect was synonymous (70.10%), followed by missense (27.44%) type (**Table 4**). We also identified, though in a reduced number of genes, effects in splice regions (0.98%) and introns (0.49%), disruptive and conservative in-frame deletions (0.49 and 0.25%, respectively), and a frameshift variant (0.25%). The impact of the polymorphisms was frequently low, which makes sense considering that most of them were predicted to have a synonymous effect. However, a polymorphism with high impact was detected. It was a 2-bp insertion that theoretically causes a frameshift mutation in the Lsat_1_v5_gn_3_1101 gene of the wild species which is responsible for the appearance of a premature stop codon. A conservative in-frame deletion was also found in this same gene. According to our results, this gene was downregulated in *L. homblei* whereas in ‘Romired’ was not differentially expressed, in which showed low expression levels in both C and DI conditions. This gene codes for a zinc-finger protein and appears in the literature as a putative TF that intervenes in the response to abiotic stress (He et al., 2020). A possible effect of one or both polymorphisms might be that the truncated protein acts as a repressor in the wild species under C conditions but stops inhibiting its target(s) as a consequence of its own downregulation under water stress. Other polymorphisms with possible important effects were the putative disruptive in-frame deletions found in Lsat_1_v5_gn_2_116640 and in Lsat_1_v5_gn_8_165301 genes, whose predicted impact was moderate. Lsat_1_v5_gn_2_116640 encodes a 70-kDa heat shock cognate protein. Heat shock proteins (HSPs) were initially described in relation to heat tolerance (Ritossa, 1962), although nowadays they are well known to be expressed in response to a great diversity of environmental stressors besides heat (reviewed in Ul Haq et al. (2019)). According to the RNA-seq analysis, this gene (Lsat_1_v5_gn_2_116640) showed a considerable increase in expression in *L. homblei* in response to drought, whereas in ‘Romired’ there was no significant change, with the values under both C and DI being similar to those in C plants of *L. homblei*. Therefore, the disruptive in-frame deletion in this gene could be inducing the activation of this HSP when *L. homblei* plants are subject to drought stress. Lsat_1_v5_gn_8_165301 encodes a Phospholipase A1 phospholipid-inositol phosphatase 2 (PLIP2) that has been described to be involved in the accumulation of anthocyanins under ABA-mediated abiotic stress responses (Wang et al., 2018). This gene also exhibited a highly significant upregulation in *L. homblei* and no change of expression in ‘Romired’. In this case, its expression levels in ‘Romired’ under C and DI conditions were similar to those of *L. homblei* under DI. Thus, the disruptive in-frame deletion found in *L. homblei* sequence might be causing the gene to be activated only under stress in the wild plants.

**Table 4:** Predicted effects for the polymorphisms detected in the 19 candidate differentially expressed genes (DEGs) in *L. homblei*.

Gene ID	Conservative in-frame deletion^a^	Disruptive in-frame deletion^a^	Frameshift variant^a^	Intron variant	Missense variant^a^	Splice region variant	Synonymous variant
Lsat_1_v5_gn_1_21441	–	–	–	–	**23**	–	27
Lsat_1_v5_gn_1_50480	–	–	–	–	**3**	–	5
Lsat_1_v5_gn_1_109200	–	–	–	–	**4**	–	27
Lsat_1_v5_gn_1_127541	–	–	–	–	**1**	–	3
Lsat_1_v5_gn_2_15680	–	–	–	–	**3**	–	3
Lsat_1_v5_gn_2_43400	–	–	–	–	**5**	1	31
Lsat_1_v5_gn_2_47181	–	–	–	–	**7**	–	10
Lsat_1_v5_gn_2_90361	–	–	–	–	**2**	–	3
Lsat_1_v5_gn_2_116640	–	**1**	–	2	**4**	–	51
Lsat_1_v5_gn_3_1101	**1**	–	**1**	–	**12**	–	8
Lsat_1_v5_gn_3_20640	–	–	–	–	**4**	–	11
Lsat_1_v5_gn_5_7401	–	–	–	–	**7**	–	16
Lsat_1_v5_gn_5_10141	–	–	–	–	**1**	1	4
Lsat_1_v5_gn_5_26000	–	–	–	–	**8**	–	12
Lsat_1_v5_gn_6_67540	–	–	–	–	**5**	–	11
Lsat_1_v5_gn_7_92980	–	–	–	–	**3**	–	2
Lsat_1_v5_gn_8_157561	–	–	–	–	**3**	2	24
Lsat_1_v5_gn_8_165301	–	**1**	–	–	**13**	–	19
Lsat_1_v5_gn_9_80621	–	–	–	–	**4**	–	19
Percentage	0.25	0.49	0.25	0.49	27.44	0.98	70.10

a High and moderate effects are shown in bold.

## 4 Discussion

### 4.1 Identification and analysis of DEGs under drought stress conditions

The number of exclusive up- and downregulated genes was more than twice in *L. homblei* and 1.3 times higher in ‘Romired’, respectively. In general, genes related to regulation within biological process (e.g., response to water) and molecular function categories were more abundant and more intensively upregulated in the wild species whereas genes responsible for cellular components were more commonly and significantly upregulated in the cultivated species. In the case of the downregulated DEGs, the most represented terms in both species were those related to catalytic activities. In general terms, basal and growth-related processes were deactivated in both species, which probably contributes to redirect resources to guarantee plant survival.

Interestingly, activation of responses seemed to be species specific, and it looks like the CWR was triggering more mechanisms of response to drought stress as the number of upregulated genes was clearly larger in *L. homblei* under DI. In contrast, the higher number of downregulated DEGs common to both species, many of them implied in basal processes, could be due to the deactivation of basal metabolism processes to designate more resources to water deficit tolerance, previously described in different plant species subject to water stress (Shao et al., 2009). The results from the GO enrichment analysis are in agreement with those found in other studies that assessed different stresses in lettuce, in which response to stimulus, biological regulation, metabolic processes, binding and catalytic activities, as well as membrane components, were the most represented terms (Wang et al., 2017; Zhou et al., 2023). Interestingly, in a transcriptomic analysis carried out to identify genes involved in lettuce anthocyanin accumulation, the most represented GO terms were the same (Zhang et al., 2016).

Both anthocyanin contents and the number of upregulated genes were clearly larger in *L. homblei* under DI. Therefore, results point to a relationship between gene expression profiles (for some DEGs) and changes in the accumulation of these antioxidant compounds. In addition, not only the most abundant anthocyanins showing the biggest change in quantity in response to water stress but also the minor anthocyanins only identified under DI (peonidin 3-*O*-glucoside and cyanidin 3-(6’’-acetylglucoside) in *L. homblei* and ‘Romired’, respectively) could play a role in the response to drought. That is more plausible in the case of *L. homblei* where the differences in anthocyanin content between C and DI conditions were significant (Medina-Lozano et al., 2024).

Interestingly, to activate the anthocyanin biosynthesis route in *L. homblei* seems to be enough to upregulate the isoforms of the gene controlling the first step of the specific pathway branch (i.e., *CHS*), even when the preceding genes from the general phenylpropanoid pathway (e.g., 4-coumarate-CoA ligase (*4CL*)) could be downregulated or not significantly differentially expressed, as they are involved in the biosynthesis of many other phenylpropanoids apart from anthocyanins. Something similar has been shown in a previous study on the expression of those genes and the anthocyanin content of poplar leaves (Tian et al., 2021).

All these differences between both *Lactuca* spp. might reflect a lager plasticity of the wild species to adapt to environmental changes. The great genetic diversity of wild species allows them to counteract the effects of different stresses more effectively (Jordanovska et al., 2020), whereas the cultivated species could have lost these mechanisms through domestication. In fact, the common DEGs to both accessions which show an opposite sense in the change of expression could consist of genes that have acquired a different mode of action as *L. homblei* belongs to the lettuce tertiary gene pool, the most genetically distant from *L. sativa.* Alternatively, they could be artefacts, either methodological (e.g., library preparation) or statistical or even both.

### 4.2 Validation, putative function, and polymorphisms of candidate DEGs

The fact that the genes with the strongest change of expression and correlation with anthocyanin content and drought were differentially expressed only in *L. homblei* might reveal, once again, the existence of tolerance mechanisms in the wild species that are not present in the cultivated one. This is in agreement with the wild species showing the highest increase (and the only resulting statistically significant) of anthocyanins in a previous study on drought stress with the same accessions, among others (Medina-Lozano et al., 2024).

The reliability of the results from the RNA-seq analysis was confirmed as the candidate DEGs were validated by real-time qPCR, being all differentially expressed only in the wild species, in which the expression profiles obtained with the two techniques coincided. Besides, most gene products of the selected DEGs have been described to participate in the response to one or more types of stresses which makes sense as some stresses often occur simultaneously.

The fact that the genes with a high change in the level of expression identified in this study resulted to be related to both the stress response and the anthocyanin content could indicate that these compounds are playing an important role for plants to cope with the drought conditions, as was also proposed before in purple-stem *Brassica napus* L. (Chen et al., 2022b).

Talking about the polymorphisms found in all DEGs and in the candidate genes, our results are in agreement with studies carried out in other crops that also used transcriptomic data, in which the number of SNPs was also much higher than the number of indels, and the most abundant polymorphism effects were synonymous and missense variants too (Iquebal et al., 2017; Muñoz-Espinoza et al., 2020). The impact of the polymorphisms identified in the candidate DEGs frequently resulted low as most of them were predicted to have a synonymous effect. However, a few of them showed a high or moderate predicted impact what could be a reflection of the drastic changes in the gene expression profiles (either activation or inhibition) in the wild species when subject to drought stress. Among the rest of the polymorphisms found, the missense variants could have an impact on the function of the resultant protein, though this one has been predicted to be moderate.

*In silico* tools are truly useful for obtaining information of functional and structural variants on the transcriptome and their possible correlation with phenotypic changes (Yazar and Özbek, 2021). However, further experimental approaches like functional analyses are essential to verify in the future the polymorphism effect found in putative candidate genes involved in the anthocyanin accumulation and the response to drought stress.

## 5 Conclusion

Mechanisms of response to drought stress related to anthocyanins were triggered in the wild species *L. homblei* but not in the cultivated lettuce variety ‘Romired’. The involvement of the proposed candidate genes in the increase of anthocyanin content and the response to drought stress in the wild species is supported by their large and significant changes in the expression levels when the plants were subjected to water deprivation and by their high correlation with anthocyanin content. Furthermore, the activation of the anthocyanin biosynthesis route was mainly achieved by significantly upregulating the genes controlling the first step of the specific branch (flavonoid pathway), again exclusively in the wild species.

All the candidate genes have been reported before to be involved in the response to biotic or abiotic stresses in other species (but not in lettuce), what demonstrate that plants have developed interconnected and interacting routes to deploy integrated responses to combinations of concurrent stresses.

This wild species has become a potential donor of drought tolerance genes to the cultivated lettuce that foreseeably will make the crop more resilient and sustainable, while containing more beneficial compounds (i.e., anthocyanins) for human health.

## Data Availability

The datasets presented in this study can be found in the European Nucleotide Archive (ENA) at https://www.ebi.ac.uk/ena/browser/home accession number: PRJEB75159.

## Supplementary material

Supplementary Table 1 Primer sequences, amplicon length, and annealing temperature of the reference gene (*TRXL3-3*) and the 19 differentially expressed genes (DEGs) selected to be validated by qPCR.

Supplementary Table 2 Predicted effects for the polymorphisms detected in the 19 candidate differentially expressed genes (DEGs) in ‘Romired’.

Supplementary Figure 1 Expression data obtained by qPCR (relative expression) and by RNA-seq (CPM, counts per million) of 19 selected genes in the lettuce commercial variety ‘Romired’ under control (C) and deficit irrigation (DI) conditions. Bars represent standard error of the mean (n=3). Transformation 1/(1+x)^2^ was applied to achieve normal distribution in qPCR data in the following cases: Lsat_1_v5_gn_1_21441, Lsat_1_v5_gn_2_43400, Lsat_1_v5_gn_2_90361, and Lsat_1_v5_gn_6_67540. Wilcoxon test was used with non-normally distributed qPCR data of Lsat_1_v5_gn_1_127541, Lsat_1_v5_gn_2_15680, Lsat_1_v5_gn_2_47181, Lsat_1_v5_gn_3_1101, Lsat_1_v5_gn_3_20640, and Lsat_1_v5_gn_5_10141.

Supplementary Figure 2 Heatmap representation of hierarchical analysis of the expression data of 19 selected differentially expressed genes (DEGs) in *L. homblei* and ‘Romired’ under control (C) and deficit irrigation (DI) conditions according to (A) RNA-seq and (B) real-time qPCR analyses. Numbers 1-3 and 4-6 represent the biological replicates under C and DI, respectively.

## References

[B1] Becker C. Klaering H. P. Kroh L. W. Krumbein A. (2014). Cool-cultivated red leaf lettuce accumulates cyanidin-3-*O*-(6″-*O*- malonyl)-glucoside and caffeoylmalic acid. Food Chem. 146, 404–411. doi: 10.1016/j.foodchem.2013.09.061 24176360

[B2] Bhattarai S. Fu Y. B. Coulman B. Tanino K. Karunakaran C. Biligetu B. (2021). Transcriptomic analysis of differentially expressed genes in leaves and roots of two alfalfa (*Medicago sativa* L.) cultivars with different salt tolerance. BMC Plant Biol. 21, 446. doi: 10.1186/s12870-021-03201-4 34610811 PMC8491396

[B3] Bolger A. M. Lohse M. Usadel B. (2014). Trimmomatic: a flexible trimmer for Illumina sequence data. Bioinformatics 30, 2114–2120. doi: 10.1093/bioinformatics/btu170 24695404 PMC4103590

[B4] Cao Y. Mei Y. Zhang R. Zhong Z. Yang X. Xu C.. (2024). Transcriptional regulation of flavonol biosynthesis in plants. Hortic. Res. 11, uhae043. doi: 10.1093/hr/uhae043 38623072 PMC11017525

[B5] Castellarin S. D. Pfeiffer A. Sivilotti P. Degan M. Peterlunger E. Di Gaspero G. (2007). Transcriptional regulation of anthocyanin biosynthesis in ripening fruits of grapevine under seasonal water deficit. Plant Cell Environ. 30, 1381–1399. doi: 10.1111/j.1365-3040.2007.01716.x 17897409

[B6] Chen L. Xu M. Liu C. Hao J. Fan S. Han Y. (2022a). LsMYB15 regulates bolting in leaf lettuce (*Lactuca sativa* L.) under high-temperature stress. Front. Plant Sci. 13. doi: 10.3389/fpls.2022.921021 PMC9275828 35837450

[B7] Chen W. Miao Y. Ayyaz A. Hannan F. Huang Q. Ulhassan Z.. (2022b). Purple stem *Brassica napus* exhibits higher photosynthetic efficiency, antioxidant potential and anthocyanin biosynthesis related genes expression against drought stress. Front. Plant Sci. 13. doi: 10.3389/fpls.2022.936696 PMC9366039 35968110

[B8] Cingolani P. Platts A. Wang L. L. Coon M. Nguyen T. Wang L.. (2012). A program for annotating and predicting the effects of single nucleotide polymorphisms, SnpEff. Fly 6, 80–92. doi: 10.4161/fly.19695 22728672 PMC3679285

[B9] Clément M. Leonhardt N. Droillard M.-J. Reiter I. Montillet J.-L. Genty B.. (2011). The cytosolic/nuclear HSC70 and HSP90 molecular chaperones are important for stomatal closure and modulate abscisic acid-dependent physiological responses in *Arabidopsis* . Plant Physiol. 156, 1481–1492. doi: 10.1104/pp.111.174425 21586649 PMC3135925

[B10] Comella P. Pontvianne F. Lahmy S. Vignols F. Barbezier N. DeBures A.. (2008). Characterization of a ribonuclease III-like protein required for cleavage of the pre-rRNA in the 3′ETS in *Arabidopsis* . Nucleic Acids Res. 36, 1163–1175. doi: 10.1093/nar/gkm1130 18158302 PMC2275086

[B11] Dangel N. J. Knoll A. Puchta H. (2014). MHF1 plays Fanconi anaemia complementation group M protein (FANCM)-dependent and FANCM-independent roles in DNA repair and homologous recombination in plants. Plant J. 78, 822–833. doi: 10.1111/tpj.12507 24635147

[B12] Eriksen R. L. Knepper C. Cahn M. D. Mou B. (2016). Screening of lettuce germplasm for agronomic traits under low water conditions. HortScience 51, 669–679. doi: 10.21273/hortsci.51.6.669

[B13] Everaert C. Luypaert M. Maag J. L. V. Cheng Q. X. Dinger M. E. Hellemans J.. (2017). Benchmarking of RNA-sequencing analysis workflows using whole-transcriptome RT-qPCR expression data. Sci. Rep. 7, 1559. doi: 10.1038/s41598-017-01617-3 28484260 PMC5431503

[B14] FAO. (2021). The impact of disasters and crises on agriculture and food security: 2021 (Rome, Italy). doi: 10.4060/cb3673en

[B15] FAOSTAT. (2021). Statistics of the Food and Agriculture Organization of the United Nations. Available online at: http://www.fao.org/faostat/en/data/QC (Accessed January 10, 2024).

[B16] Faria D. (2017). GOEnrichment (GitHub Repos). Available online at: https://github.com/DanFaria/GOEnrichment (Accessed February 10, 2024).

[B17] Farooq M. Wahid A. Kobayashi N. Fujita D. Basra S. M. A. (2009). “Plant drought stress: effects, mechanisms and management,” in Sustainable Agriculture. Eds. Lichtfouse E. Navarrete M. Debaeke P. Véronique S. Alberola C. (Springer, Dordrecht), 153–188. doi: 10.1007/978-90-481-2666-8_12

[B18] Fujimori N. Suzuki N. Nakajima Y. Suzuki S. (2014). Plant DNA-damage repair/toleration 100 protein repairs UV-B-induced DNA damage. DNA Repair. 21, 171–176. doi: 10.1016/j.dnarep.2014.05.009 24951183

[B19] Garcia C. Blesso C. N. (2021). Antioxidant properties of anthocyanins and their mechanism of action in atherosclerosis. Free Radic. Biol. Med. 172, 152–166. doi: 10.1016/j.freeradbiomed.2021.05.040 34087429

[B20] Garrison E. P. (2015).vcflib. In: GitHub repository (GitHub). Available online at: https://github.com/ekg/vcflib (Accessed February 10, 2024).

[B21] Garrison E. P. Marth G. T. (2012). Haplotype-based variant detection from short-read sequencing (Ithaca, United States: Cornell University). Available at: http://arxiv.org/abs/1207.3907 (Accessed February 09, 2024).

[B22] He F. Niu M. X. Feng C. H. Li H. G. Su Y. Su W. L.. (2020). *PeSTZ1* confers salt stress tolerance by scavenging the accumulation of ROS through regulating the expression of *PeZAT12* and *PeAPX2* in *Populus* . Tree Physiol. 40, 1292–1311. doi: 10.1093/treephys/tpaa050 32334430

[B23] Horvath S. Dong J. (2008). Geometric interpretation of gene coexpression network analysis. PloS Comput. Biol. 4, e1000117. doi: 10.1371/journal.pcbi.1000117 18704157 PMC2446438

[B24] Hugueney P. Provenzano S. Verriès C. Ferrandino A. Meudec E. Batelli G.. (2009). A novel cation-dependent *O*-methyltransferase involved in anthocyanin methylation in grapevine. Plant Physiol. 150, 2057–2070. doi: 10.1104/pp.109.140376 19525322 PMC2719152

[B25] Ino I. Yamaguchi M. A. (1993). Acetyl-coenzyme A: Anthocyanidin 3-glucoside acetyltransferase from flowers of *Zinnia elegans* . Phytochemistry 33, 1415–1417. doi: 10.1016/0031-9422(93)85101-V

[B26] IPCC (2021). “Summary for policymakers,” in Climate Change 2021. The Physical Science Basis. Contribution of Working Group I to the Sixth Assessment Report of the Intergovernmental Panel on Climate Change, vol. Vol. 2 . Eds. Masson-Delmotte V. Zhai P. Pirani A. (Cambridge, United Kingdom: Cambridge University Press).

[B27] Iquebal M. A. Soren K. R. Gangwar P. Shanmugavadivel P. S. Aravind K. Singla D.. (2017). Discovery of putative herbicide resistance genes and its regulatory network in chickpea using transcriptome sequencing. Front. Plant Sci. 8. doi: 10.3389/fpls.2017.00958 PMC5461349 28638398

[B28] Jia J. Ji R. Li Z. Yu Y. Nakano M. Long Y.. (2020). Soybean DICER-LIKE2 regulates seed coat color via production of primary 22-nucleotide small interfering RNAs from long inverted repeats. Plant Cell 32, 3662–3673. doi: 10.1105/tpc.20.00562 33077493 PMC7721327

[B29] Jordanovska S. Jovovic Z. Andjelkovic V. (2020). “Potential of wild species in the scenario of climate change,” in Rediscovery of Genetic and Genomic Resources for Future Food Security. Eds. Salgotra R. K. Zargar S. M. (Springer Nature, Singapore), 263–302. doi: 10.1007/978-981-15-0156-2_10

[B30] Ju Y. Yang B. He S. Tu T. Min Z. Fang Y.. (2019). Anthocyanin accumulation and biosynthesis are modulated by regulated deficit irrigation in Cabernet Sauvignon (*Vitis vinifera* L.) grapes and wines. Plant Physiol. Biochem. 135, 469–479. doi: 10.1016/j.plaphy.2018.11.013 30473422

[B31] Kim D. Langmead B. Salzberg S. L. (2015). HISAT: a fast spliced aligner with low memory requirements. Nat. Methods 12, 357–360. doi: 10.1038/nmeth.3317 25751142 PMC4655817

[B32] Koyama R. Yoshimoto A. Ishibashi M. Itoh H. Uno Y. (2021). Enzymatic activities and gene transcript levels associated with the augmentation of antioxidant constituents during drought stress in lettuce. Horticulturae 7, 444. doi: 10.3390/horticulturae7110444

[B33] Langfelder P. Horvath S. (2008). WGCNA: An R package for weighted correlation network analysis. BMC Bioinf. 9, 559. doi: 10.1186/1471-2105-9-559 PMC2631488 19114008

[B34] Lee S. Choi E. Kim T. Hwang J. Lee J.-H. (2022). AtHAD1, A haloacid dehalogenase-like phosphatase, is involved in repressing the ABA response. Biochem. Biophys. Res. Commun. 587, 119–125. doi: 10.1016/j.bbrc.2021.11.095 34871999

[B35] Li M. Liu J. Zhou Y. Zhou S. Zhang S. Tong H.. (2020). Transcriptome and metabolome profiling unveiled mechanisms of tea (*Camellia sinensis*) quality improvement by moderate drought on pre-harvest shoots. Phytochemistry 180, 112515. doi: 10.1016/j.phytochem.2020.112515 32957017

[B36] Liao Y. Smyth G. K. Shi W. (2013). featureCounts: an efficient general purpose program for assigning sequence reads to genomic features. Bioinformatics 30, 923–930. doi: 10.1093/bioinformatics/btt656 24227677

[B37] Llorach R. Martínez-Sánchez A. Tomás-Barberán F. A. Gil M. I. Ferreres F. (2008). Characterisation of polyphenols and antioxidant properties of five lettuce varieties and escarole. Food Chem. 108, 1028–1038. doi: 10.1016/j.foodchem.2007.11.032 26065768

[B38] Medina-Lozano I. Arnedo M. S. Grimplet J. Díaz A. (2023). Selection of novel reference genes by RNA-seq and their evaluation for normalising real-time qPCR expression data of anthocyanin-related genes in lettuce and wild relatives. Int. J. Mol. Sci. 24, 3052. doi: 10.3390/ijms24033052 36769376 PMC9917471

[B39] Medina-Lozano I. Bertolín J. R. Díaz A. (2021). Nutritional value of commercial and traditional lettuce (*Lactuca sativa* L.) and wild relatives: vitamin C and anthocyanin content. Food Chem. 359, 129864. doi: 10.1016/j.foodchem.2021.129864 33962194

[B40] Medina-Lozano I. Bertolín J. R. Díaz A. (2024). Impact of drought stress on vitamin C and anthocyanin content in cultivated lettuces (*Lactuca sativa* L.) and wild relatives (*Lactuca* spp.). Front. Plant Sci. 15. doi: 10.3389/fpls.2024.1369658 PMC10983614 38562559

[B41] Moreno-Escamilla J. O. Jiménez-Hernández F. E. Alvarez-Parrilla E. de la Rosa L. A. Martínez-Ruiz N. D. R. González-Fernández R.. (2020). Effect of Elicitation on Polyphenol and Carotenoid Metabolism in Butterhead Lettuce (*Lactuca sativa* var. capitata). ACS Omega 5, 11535–11546. doi: 10.1021/acsomega.0c00680 32478243 PMC7254786

[B42] Mou B. (2005). Genetic variation of beta-carotene and lutein contents in lettuce. J. Am. Soc Hortic. Sci. 130, 870–876. doi: 10.21273/jashs.130.6.870

[B43] Muñoz-Espinoza C. Di Genova A. Sánchez A. Correa J. Espinoza A. Meneses C.. (2020). Identification of SNPs and InDels associated with berry size in table grapes integrating genetic and transcriptomic approaches. BMC Plant Biol. 20, 1–21. doi: 10.1186/s12870-020-02564-4 32746778 PMC7397606

[B44] Na J.-K. Metzger J. D. (2020). A putative tomato inositol polyphosphate 5-phosphatase, Le5PT1, is involved in plant growth and abiotic stress responses. 3 Biotech. 10, 28. doi: 10.1007/s13205-019-2023-y PMC6942557 31950007

[B45] Naing A. H. Kim C. K. (2021). Abiotic stress-induced anthocyanins in plants: Their role in tolerance to abiotic stresses. Physiol. Plant 172, 1711–1723. doi: 10.1111/ppl.13373 33605458

[B46] Paim B. T. Crizel R. L. Tatiane S. J. Rodrigues V. R. Rombaldi C. V. Galli V. (2020). Mild drought stress has potential to improve lettuce yield and quality. Sci. Hortic. (Amsterdam) 272, 109578. doi: 10.1016/j.scienta.2020.109578

[B47] Palusa S. G. Golovkin M. Shin S. B. Richardson D. N. Reddy A. S. N. (2007). Organ-specific, developmental, hormonal and stress regulation of expression of putative pectate lyase genes in *Arabidopsis* . New Phytol. 174, 537–550. doi: 10.1111/j.1469-8137.2007.02033.x 17447910

[B48] Park S. Shi A. Mou B. (2020). Genome-wide identification and expression analysis of the CBF/DREB1 gene family in lettuce. Sci. Rep. 10, 5733. doi: 10.1038/s41598-020-62458-1 32235838 PMC7109083

[B49] Pfaffl M. W. (2001). A new mathematical model for relative quantification in real-time RT-PCR. Nucleic Acids Res. 29, e45. doi: 10.1093/nar/29.9.e45 11328886 PMC55695

[B50] PGR (Plant Genetic Resources) Lettuce. The lettuce gene pool. Available online at: https://www.pgrportal.nl/en/lettuce-genetic-resources-portal.htm (Accessed January 27, 2024).

[B51] Qu J. Kang S. G. Hah C. Jang J. C. (2016). Molecular and cellular characterization of GA-Stimulated Transcripts GASA4 and GASA6 in *Arabidopsis thaliana* . Plant Sci. 246, 1–10. doi: 10.1016/j.plantsci.2016.01.009 26993231

[B52] Quezada-Martinez D. Addo Nyarko C. P. Schiessl S. V. Mason A. S. (2021). Using wild relatives and related species to build climate resilience in *Brassica* crops. Theor. Appl. Genet. 134, 1711–1728. doi: 10.1007/s00122-021-03793-3 33730183 PMC8205867

[B53] Ramírez V. López A. Mauch-Mani B. Gil M. J. Vera P. (2013). An extracellular subtilase switch for immune priming in *Arabidopsis* . PloS Pathog. 9, e1003445. doi: 10.1371/journal.ppat.1003445 23818851 PMC3688555

[B54] Reddy A. R. Chaitanya K. V. Vivekanandan M. (2004). Drought-induced responses of photosynthesis and antioxidant metabolism in higher plants. J. Plant Physiol. 161, 1189–1202. doi: 10.1016/j.jplph.2004.01.013 15602811

[B55] Reyes-Chin-Wo S. Wang Z. Yang X. Kozik A. Arikit S. Song C.. (2017). Genome assembly with *in vitro* proximity ligation data and whole-genome triplication in lettuce. Nat. Commun. 8, 14953. doi: 10.1038/ncomms14953 28401891 PMC5394340

[B56] Ritossa F. (1962). A new puffing pattern induced by temperature shock and DNP in drosophila. Experientia 18, 571–573. doi: 10.1007/BF02172188

[B57] Robinson M. D. McCarthy D. J. Smyth G. K. (2009). edgeR: a Bioconductor package for differential expression analysis of digital gene expression data. Bioinformatics 26, 139–140. doi: 10.1093/bioinformatics/btp616 19910308 PMC2796818

[B58] Rugienius R. Bendokas V. Siksnianas T. Stanys V. Sasnauskas A. Kazanaviciute V. (2021). Characteristics of *Fragaria vesca* Yield Parameters and Anthocyanin Accumulation under Water Deficit Stress. Plants 10, 557. doi: 10.3390/plants10030557 33809648 PMC8001689

[B59] Shao H. B. Chu L. Y. Jaleel C. A. Manivannan P. Panneerselvam R. Shao M. A. (2009). Understanding water deficit stress-induced changes in the basic metabolism of higher plants-biotechnologically and sustainably improving agriculture and the ecoenvironment in arid regions of the globe. Crit. Rev. Biotechnol. 29, 131–151. doi: 10.1080/07388550902869792 19412828

[B60] Shim J. S. Oh N. Chung P. J. Kim Y. S. Choi Y. D. Kim J. K. (2018). Overexpression of OsNAC14 improves drought tolerance in rice. Front. Plant Sci. 9. doi: 10.3389/fpls.2018.00310 PMC5855183 29593766

[B61] Su W. Tao R. Liu W. Yu C. Yue Z. He S.. (2020). Characterization of four polymorphic genes controlling red leaf colour in lettuce that have undergone disruptive selection since domestication. Plant Biotechnol. J. 18, 479–490. doi: 10.1111/pbi.13213 31325407 PMC6953203

[B62] Tahmasebi A. Ashrafi-Dehkordi E. Shahriari A. G. Mazloomi S. M. Ebrahimie E. (2019). Integrative meta-analysis of transcriptomic responses to abiotic stress in cotton. Prog. Biophys. Mol. Biol. 146, 112–122. doi: 10.1016/j.pbiomolbio.2019.02.005 30802474

[B63] Tange O. (2011). GNU Parallel: The Command-Line Power Tool Vol. 36 (Frederiksberg, Denmark: Login USENIX Mag), 42–47. Available at: https://www.gnu.org/software/parallel/. (Accessed February 09, 2024)

[B64] The Galaxy Community (2022). The Galaxy platform for accessible, reproducible and collaborative biomedical analyses: 2022 update. Nucleic Acids Res. 50, W345–W351. doi: 10.1093/nar/gkac247 35446428 PMC9252830

[B65] Tian Y. Li Q. Rao S. Wang A. Zhang H. Wang L.. (2021). Metabolic profiling and gene expression analysis provides insights into flavonoid and anthocyanin metabolism in poplar. Tree Physiol. 41, 1046–1064. doi: 10.1093/treephys/tpaa152 33169130

[B66] Tsitsekian D. Daras G. Templalexis D. Avgeri F. Lotos L. Orfanidou C. G.. (2023). A subset of highly responsive transcription factors upon tomato infection by pepino mosaic virus. Plant Biol. 25, 529–540. doi: 10.1111/plb.13515 36856454

[B67] Tsormpatsidis E. Henbest R. G. C. Davis F. J. Battey N. H. Hadley P. Wagstaffe A. (2008). UV irradiance as a major influence on growth, development and secondary products of commercial importance in Lollo Rosso lettuce “Revolution” grown under polyethylene films. Environ. Exp. Bot. 63, 232–239. doi: 10.1016/j.envexpbot.2007.12.002

[B68] Ul Haq S. Khan A. Ali M. Khattak A. M. Gai W.-X. Zhang H.-X.. (2019). Heat shock proteins: dynamic biomolecules to counter plant biotic and abiotic stresses. Int. J. Mol. Sci. 20, 5321. doi: 10.3390/ijms20215321 31731530 PMC6862505

[B69] Wada K. C. Inagaki N. Sakai H. Yamashita H. Nakai Y. Fujimoto Z.. (2022). Genetic effects of Red Lettuce Leaf genes on red coloration in leaf lettuce under artificial lighting conditions. Plant Environ. Interact. 3, 179–192. doi: 10.1002/pei3.10089 37283610 PMC10168059

[B70] Wang Y. Chen R. Hao Y. Liu H. Song S. Sun G. (2017). Transcriptome analysis reveals differentially expressed genes (DEGs) related to lettuce (*Lactuca sativa*) treated by TiO2/ZnO nanoparticles. Plant Growth Regul. 83, 13–25. doi: 10.1007/s10725-017-0280-5

[B71] Wang Z. Gerstein M. Snyder M. (2009). RNA-Seq: a revolutionary tool for transcriptomics. Nat. Rev. Genet. 10, 57–63. doi: 10.1038/nrg2484 19015660 PMC2949280

[B72] Wang K. Guo Q. Froehlich J. E. Hersh H. L. Zienkiewicz A. Howe G. A.. (2018). Two abscisic acid-responsive plastid lipase genes involved in jasmonic acid biosynthesis in *Arabidopsis thaliana* . Plant Cell 30, 1006–1022. doi: 10.1105/tpc.18.00250 29666162 PMC6002186

[B73] Wei Z. Hu K. Zhao D. L. Tang J. Huang Z. Q. Jin P.. (2020). MYB44 competitively inhibits the formation of the MYB340-bHLH2-NAC56 complex to regulate anthocyanin biosynthesis in purple-fleshed sweet potato. BMC Plant Biol. 20, 258. doi: 10.1186/s12870-020-02451-y 32503504 PMC7275474

[B74] Wickham H. (2009). ggplot2 - Elegant Graphics for Data Analysis (New York, NY, USA: Springer). doi: 10.1007/978-3-319-24277-4

[B75] Wu N. Li W. J. Chen C. Zhao Y. P. Hou Y. X. (2022). Analysis of the PRA1 genes in cotton identifies the role of GhPRA1.B1-1A in *Verticillium dahliae* resistance. Genes (Basel) 13, 765. doi: 10.3390/genes13050765 35627150 PMC9141244

[B76] Xiong T. Zhang S. Kang Z. Zhang T. Li S. (2021). Dose-Dependent Physiological and Transcriptomic Responses of Lettuce (*Lactuca sativa* L.) to Copper Oxide Nanoparticles-Insights into the Phytotoxicity Mechanisms. Int. J. Mol. Sci. 22, 3688. doi: 10.3390/ijms22073688 33916236 PMC8036535

[B77] Yan H. Pei X. Zhang H. Li X. Zhang X. Zhao M.. (2021). MYB-mediated regulation of anthocyanin biosynthesis. Int. J. Mol. Sci. 22, 3103. doi: 10.3390/ijms22063103 33803587 PMC8002911

[B78] Yazar M. Özbek P. (2021). *In silico* tools and approaches for the prediction of functional and structural effects of single-nucleotide polymorphisms on proteins: an expert review. Omi. A J. Integr. Biol. 25, 23–37. doi: 10.1089/omi.2020.0141 33058752

[B79] Zeljković S. C. Štefelová N. Hron K. Doležalová I. Tarkowski P. (2023). Preharvest abiotic stress affects the nutritional value of lettuce. Agronomy 13, 398. doi: 10.3390/agronomy13020398

[B80] Zhang F. Rosental L. Ji B. Brotman Y. Dai M. (2024). Metabolite-mediated adaptation of crops to drought and the acquisition of tolerance. Plant J. 118, 626–644. doi: 10.1111/tpj.16634 38241088

[B81] Zhang H. Wan Z. Liu J. Hu X. Ren L. Feng S.. (2022). DsCER26 affects the leaf dehydration tolerance of rice by altering cuticular wax alkane production without affecting the grain fatty acid content. ACS Agric. Sci. Technol. 2, 813–822. doi: 10.1021/acsagscitech.2c00141

[B82] Zhang Y. Xu S. Cheng Y. Peng Z. Han J. (2018). Transcriptome profiling of anthocyanin-related genes reveals effects of light intensity on anthocyanin biosynthesis in red leaf lettuce. PeerJ 13, e4607. doi: 10.7717/peerj.4607 PMC5900932 29666761

[B83] Zhang Y. Z. Xu S. Z. Cheng Y. W. Ya H. Y. Han J. M. (2016). Transcriptome analysis and anthocyanin-related genes in red leaf lettuce. Genet. Mol. Res. 15, gmr.15017023. doi: 10.4238/gmr.15017023 26909931

[B84] Zhang B. Zhu Z. Z. Qu D. Wang B. C. Hao N. N. Yang Y. Z.. (2021). MdBBX21, a B-box protein, positively regulates light-induced anthocyanin accumulation in apple peel. Front. Plant Sci. 12. doi: 10.3389/fpls.2021.774446 PMC8633397 34868172

[B85] Zhou H. Yu L. Liu S. Zhu A. Yang Y. Chen C.. (2023). Transcriptome comparison analyses in UV-B induced AsA accumulation of *Lactuca sativa* L. BMC Genomics 24, 61. doi: 10.1186/s12864-023-09133-7 36737693 PMC9896689

[B86] Zhou T. Yue C. Huang J. Cui J. Liu Y. Wang W.. (2021). Genome-wide identification of the amino acid permease genes and molecular characterization of their transcriptional responses to various nutrient stresses in allotetraploid rapeseed. BMC Plant Biol. 21, 151. doi: 10.1186/s12870-021-03043-0 32268885 PMC7140331

[B87] Zhu H. Xia R. Zhao B. An Y. Dardick C. D. Callahan A. M.. (2012). Unique expression, processing regulation, and regulatory network of peach (*Prunus persica*) miRNAs. BMC Plant Biol. 12, 149. doi: 10.1186/1471-2229-12-149 22909020 PMC3542160

