# Supplementary figures and images for: Harnessing the diversity of a lettuce wild relative to identify anthocyanin-related genes transcriptionally responsive to drought stress
Source: Front Plant Sci. 2025 Jan 15;15:1494339. doi: 10.3389/fpls.2024.1494339 (PMC11795315; doi:10.3389/fpls.2024.1494339)

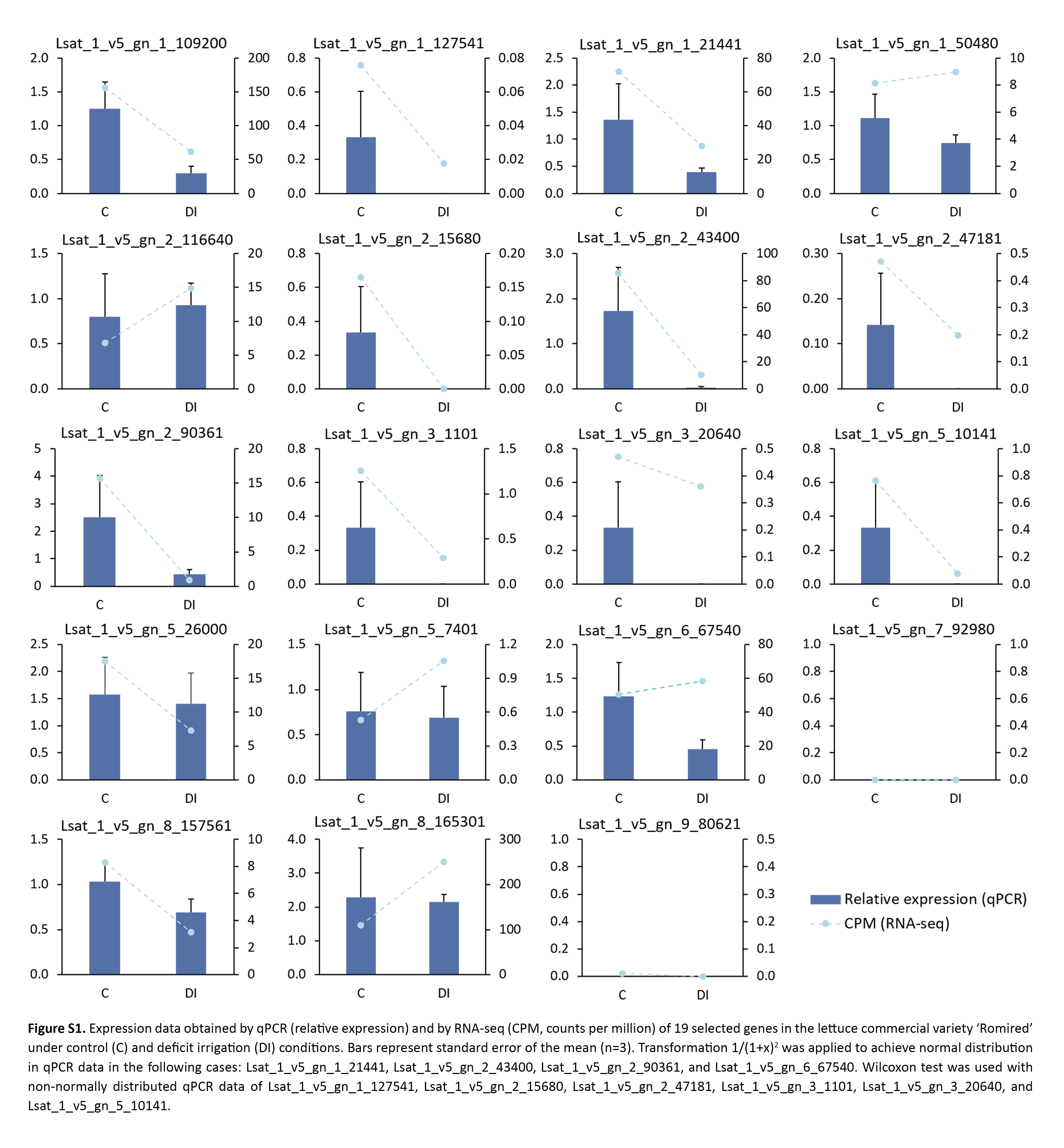

Supplement: Supplementary Figure 1 — Expression data obtained by qPCR (relative expression) and by RNA-seq (CPM, counts per million) of 19 selected genes in the lettuce commercial variety ‘Romired’ under control (C) and deficit irrigation (DI) conditions. Bars represent standard error of the mean (n=3). Transformation 1/(1+x)2 was applied to achieve normal distribution in qPCR data in the following cases: Lsat_1_v5_gn_1_21441, Lsat_1_v5_gn_2_43400, Lsat_1_v5_gn_2_90361, and Lsat_1_v5_gn_6_67540. Wilcoxon test was used with non-normally distributed qPCR data of Lsat_1_v5_gn_1_127541, Lsat_1_v5_gn_2_15680, Lsat_1_v5_gn_2_47181, Lsat_1_v5_gn_3_1101, Lsat_1_v5_gn_3_20640, and Lsat_1_v5_gn_5_10141. [file Image1.tif]

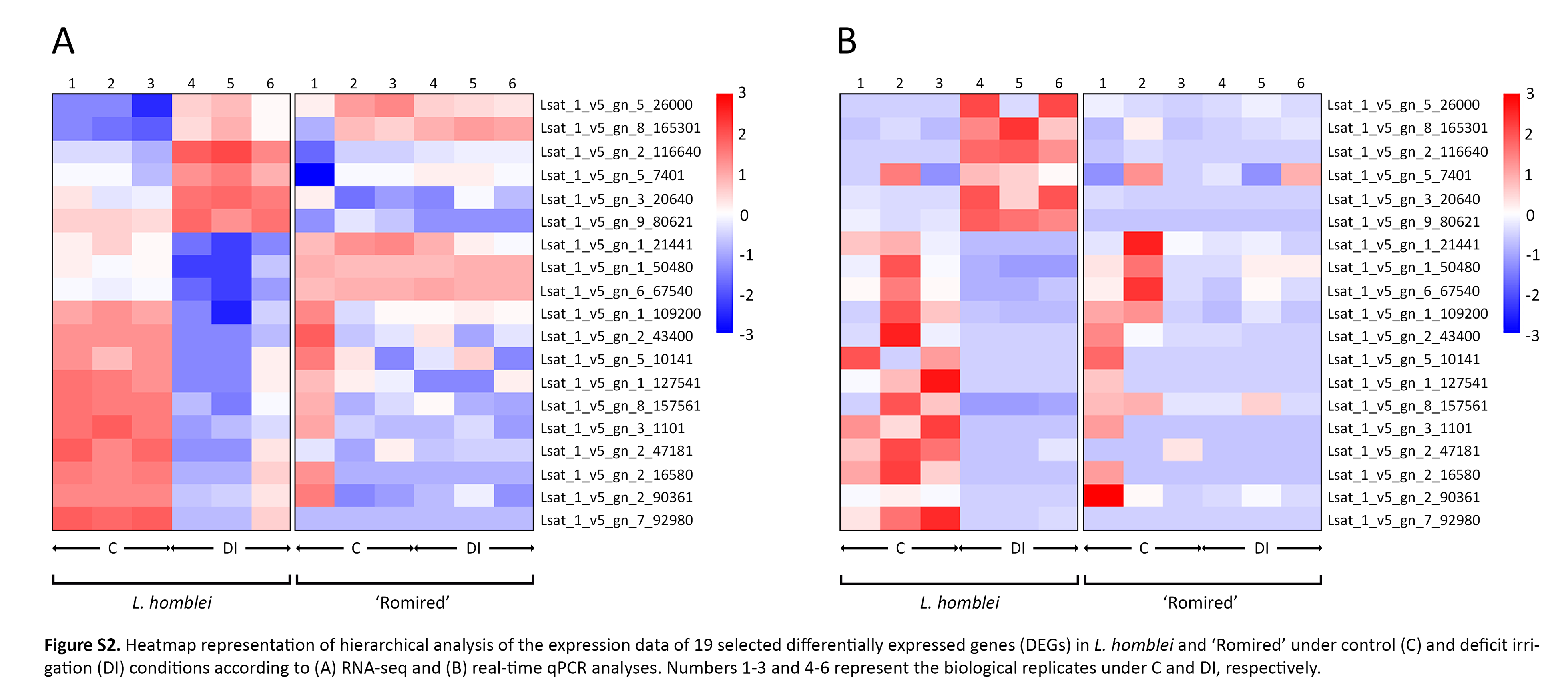

Supplement: Supplementary Figure 2 — Heatmap representation of hierarchical analysis of the expression data of 19 selected differentially expressed genes (DEGs) in L. homblei and ‘Romired’ under control (C) and deficit irrigation (DI) conditions according to (A) RNA-seq and (B) real-time qPCR analyses. Numbers 1-3 and 4-6 represent the biological replicates under C and DI, respectively. [file Image2.tif]
